# The complexometric behavior of selected aroyl-*S*,*N*-ketene acetals shows that they are more than AIEgens

**DOI:** 10.1038/s41598-024-62100-4

**Published:** 2024-05-31

**Authors:** Lukas Biesen, Thomas J. J. Müller

**Affiliations:** 1 Heinrich-Heine-Universität Düsseldorf, Math.-Nat. Fakultät, Institut für Organische Chemie und Makromolekulare Chemie, Universitätsstraße 1, 40225 Düsseldorf, Germany; 2 School of Chemistry, Joseph Black Building, University of Glasgow, Glasgow, G12 8QQ UK

**Keywords:** Coordination chemistry, Organic chemistry, Fluorescent probes

## Abstract

Using the established synthetic methods, aroyl-*S*,*N*-ketene acetals and subsequent bi- and multichromophores can be readily synthesized. Aside from pronounced AIE (aggregation induced emission) properties, these selected examples possess distinct complexometric behavior for various metals purely based on the underlying structural motifs. This affects the fluorescence properties of the materials which can be readily exploited for metal ion detection and for the formation of different metal-aroyl-*S*,*N*-ketene acetal complexes that were confirmed by Job plot analysis. In particular, gold(I), iron(III), and ruthenium (III) ions reveal complexation enhanced or quenched emission. For most dyes, weakly coodinating complexes were observed, only in case of a phenanthroline aroyl-*S*,*N*-ketene acetal multichromophore, measurements indicate the formation of a strongly coordinating complex. For this multichromophore, the complexation results in a loss of fluorescence intensity whereas for dimethylamino-aroyl-*S*,*N*-ketene acetals and bipyridine bichromophores, the observed quantum yield is nearly tripled upon complexation. Even if no stable complexes are formed, changes in absorption and emission properties allow for a simple ion detection.

## Introduction

Small traces of metal ions are ubiquitous. They can be found in nearly everything, i.e. in potable water, in nutriments, and in solvents for chemical reactions^1,2^. And while specific metals are essential for our survival and our health^3,4^, others pose a great danger due to high toxicity^5–7^. Yet, even essential metal ions can be as well toxic at elevated concentration^3,8–10^. Therefore, the precise detection of metal ions in analytics^11^ as well as in therapy and diagnostics^12^ is of utmost importance. The requirements are quite rigorous with respect to detection limit and selectivity^13,14^. Electrochemistry and engineering provided various devices to ensure high quality metal ion sensing^15–19^. Another discipline, which garnered lots of attention regarding metal ion sensing, is dye chemistry in which the presence of metal ions leads to a change of photophysical properties, thus, resulting in a preferential optical readout^14,20–27^. While the range of suitable dyes for metal ion sensing is broad, a common ground for the overwhelming majority are ligands with heteroatoms, as they often act as sites of coordination^28–33^. The modes of action for these metal ion sensors are as manifolded as their structural motifs and span from metal organic frameworks^34–36^ to classic fluorescent probes^37–40^ up to doped materials^20,41^ and carbon^42–44^ and quantum dots^45,46^. Due to the phenomenal impact of aggregation induced emission (AIE), AIEgens have also been implemented as promising metal sensors due to their remarkable fluorescence light up under specific circumstances^47–52^.

Since 2020, aroyl-*S*,*N*-ketene acetals quite successfully entered the stage of novel and exciting AIEgens due to their high versatility and their unusual AIE properties based on the presence of benzyl moieties^53,54^. By various expansions, the bouquet of photophysical properties for this class of compounds has been vastly expanded by exploiting the full potential of synthetic organic chemistry^53,55–59^. Aside from AIE, solid-state and dual emission, solvatochromism, and halochromism properties were unveiled for this class of compound^53,60^. One fact that has been overlooked for aroyl-*S*,*N*-ketene acetal based AIEgens so far, is the dyes’ behavior in the presence of metal ions. Due to various heteroatoms embedded into the molecular structure, the assumption of complexing behavior seemed feasible. Benzothiazole derivatives are applied in *N*-heterocyclic carbene ligands (NHCs), especially *N*-allyl-substituted derivatives have been successfully used in these respects^61–63^. Furthermore, a 2,5-bis[3-benzyl-2-methylbenzothiazole] croconaine derivative could be used as a highly sensitive and selective sensor for the detection of iron(III) ions with the naked eye^64^. Due to an identical distance between carbonyl oxygen and sulfur atom of the benzothiazole of the 2,5-bis[3-benzyl-2-methylbenzothiazole] croconaine derivative in comparison to the structural motif of the aroyl-*S*,*N*-ketene acetals, these complexing properties inspired to perform comparable complexometric studies for the aroyl-*S*,*N*-ketene acetal class of compounds. Due to the emission properties of the dimethylamino-substituted aroyl-*S*,*N*-ketene acetals and their derivates, we chose to make them the centerpiece of the present complexometric investigations.

## Results and discussion

### Synthesis

**Figure 1:**
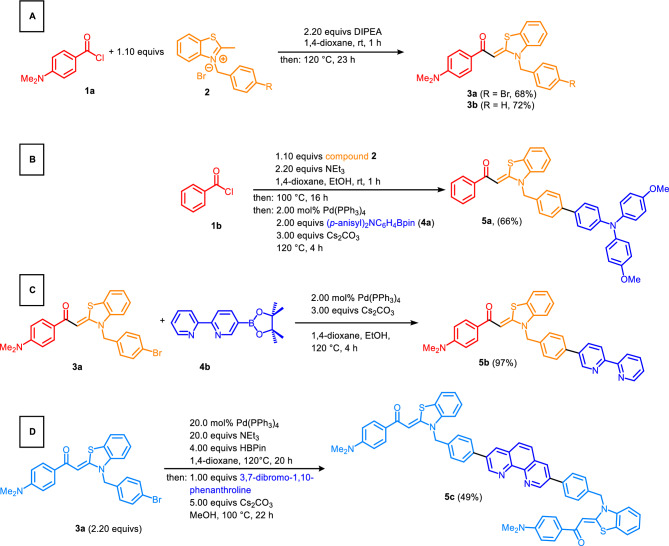
Addition–elimination reaction for the generation of dimethylamino substituted aroyl-*S*,*N*-ketene acetals **3a** and **b** (**A**), Consecutive three-component condensation-Suzuki coupling synthesis of aroyl-*S*,*N*-ketene acetal bichromophore **5a** (**B**), Suzuki coupling synthesis of dimethylamino aroyl-*S*,*N*-ketene acetal bipyridine bichromophore **5b** (**C**), MBSC synthesis of 1,10-phenanthroline bridged trichromophore **5c** (**D**).

First generation dimethylamino aroyl-*S*,*N*-ketene acetals **3a**^54^ and **3b**^54^ can be easily obtained via an addition–elimination sequence, starting from the respective acid chloride and a benzothiazolium salt **2** using diisopropylethylamine as a base in very good yields (Fig. 1A)^54^.

Another fluorescent aroyl-*S*,*N*-ketene acetal based material, which might be suitable for complexometric studies, are triphenylamine aroyl-*S*,*N*-ketene acetals that are accessible via a one-pot procedure, where the addition–elimination procedure is followed en route by a Suzuki coupling with triarylamine boronate **4a**^65^ to give the respective bichromophore **5a**^57^ in a yield of 66% (Fig. 1B)^57^. Similarly, a single step Suzuki reaction of dimethylamino aroyl-*S*,*N*-ketene acetal **3a** and bipyridine boronic acid ester **4b** leads to the formation of the respective bipyridine bichromophore **5b** in excellent yield (Fig. 1C).

By using an excess of dimethylamino aroyl-*S*,*N*-ketene acetal **3a** implemented in a Masuda borylation-Suzuki coupling (MBSC)^66^ sequence with dibrominated 1,10-phenanthroline as a coupling partner, it is possible to generate the 1,10-phenanthroline bridged dimethylamino aroyl-*S*,*N*-ketene acetal multichromophore **5c** in moderate yield (Fig. 1D)^56^.

### Photophysical properties

**Figure 2:**
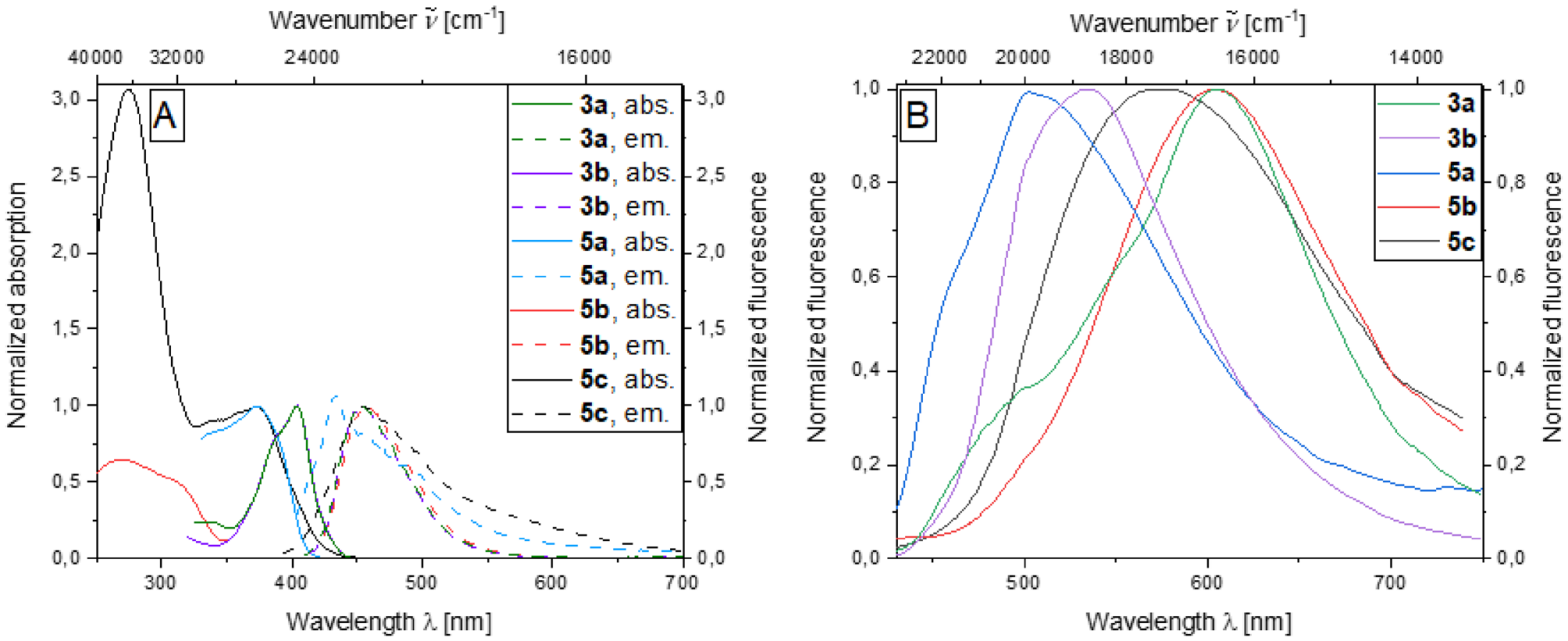
Normalized absorption and emission spectra in ethanol (*T* = 298 K, *c*(abs) = 10^−5^ m, *c*(em) = 10^−7^ m, *λ*_*ex*c_ = *λ*_*max,abs*_) (**A**) and normalized solid-state emission spectra and solid-state fluorescence colors (**B**) of aroyl-*S*,*N*-ketene acetals **3** and **5** (λ_*exc*_ = 365 nm; *λ*_*exc*_ = *λ*_*max(abs)*_).

The photophysical properties of the investigated series of dyes in ethanol are mostly determined by the aroyl-*S*,*N*-ketene acetal moiety. As most of the dyes comprise of dimethylamino aroyl-*S*,*N*-ketene acetals, for compounds **3a**, **3b**, and **5b** show nearly identical absorption properties with absorption maxima of 404 nm, only bichromophore **5a** and multichromophore **5c** deviate from this behavior due to the secondary chromophores of triphenylamine and 1,10-phenanthroline, resulting in absorption maxima of 373 nm. Regarding the luminescence in ethanol, the emission properties are determined by the dimethylamino aroyl-*S*,*N*-ketene acetal units due to their intense emission at 455 nm and fluorescence quantum yields *Φ*_*f*_ of around 0.20. Only bichromophore **5a** fluoresces at 447 nm as the unsubstituted aroyl-*S*,*N*-ketene acetal **3c** does not fluoresce in solution^54^. Therefore, the properties are determined by the secondary triphenylamine chromophore (Fig. 2A). The solid-state emission exhibits a greater range, which is determined by both the aroyl-*S*,*N*-ketene acetal and the secondary chromophore. The most blue shifted derivative is the triphenylamine bichromophore **5a** with a solid-state emission maximum of 500 nm, whereas the most bathochromically shifted emission maxima occur for both the dimethylamino bipyridine bichromophore **5b** and the brominated dimethylamino aroyl-*S*,*N*-ketene acetal **3a** with *λ*_*max,em*_ = 604 nm (Fig. 2B) (for full details of photophysical properties, see SI chpt.6).

As aggregation induced emission is normally a prime feature for aroyl-*S*,*N*-ketene acetals, we could only observe partial aggregation caused quenching effect in conjunction with a bathochromic shift of the emission maximum, which can be unequivocally attributed to the dimethylamino substituted aroyl-*S*,*N*-ketene acetal core (for details, see SI, chpt. 7).

### Complexometric studies

The standard method for determining complex stoichiometry is to use the method of continuous variation that is shown in the Job plot^67,68^. This assumes a constant total volume and concentration of all components involved in the equilibrium. The continuous variation of the ratios of metal salt and ligand concentration allows the complex stoichiometry to be determined via the change in the physical quantity under consideration. For the required Job plot, the variable photophysical quantity must be plotted against the mole fraction. It should also be noted that free binding sites in the complex can be occupied by solvent molecules if coordinating solvents are present^67,68^.

A series of qualitative tests were carried out for the dimethylamino-substituted compound **3b** by adding small amounts of various metal salts to a solution of the dye in ethanol and observing with the naked eye the influence this has on the absorption and emission properties of the dissolved chromophore. As expected, gold(I), iron(III), and ruthenium(III) salts were identified as potential candidates for complexometric studies. We screened for a variety of the most common metal salts of both transition metals and metals like sodium, potassium, magnesium, calcium, aluminum, silver, gold, iron, tin, nickel, platin, zinc and scandium ions in order to cover up the widest range possible of metals that are most commonly used and can be called ubiquitous.

**Figure 3:**
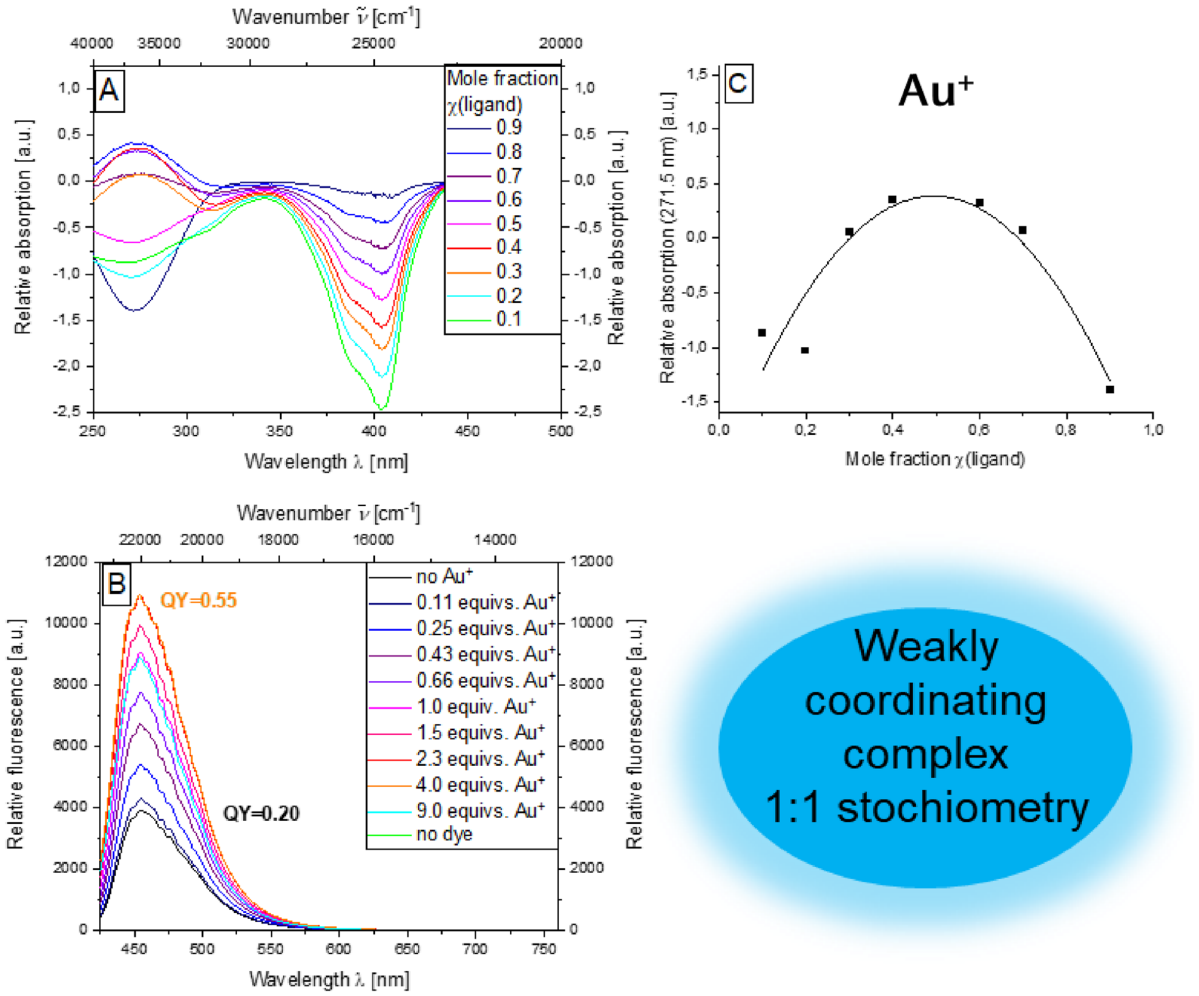
Absorption difference spectra of compound **3b** at different mole fractions of the ligand dye in the presence of AuI (recorded in ethanol; *c*(**3b**) = 10^−5^ m, *c*(metal salt) = 10^−5^ m, *T* = 298 K) (**A**), emission spectra of compound **3b** with increasing amount AuI (*c*(**3b**) = 10^−7^ m, *c*(metal salt) = 10^−7^ m, *T* = 298 K,* λ*_*exc*_ = 404 nm) (**B**), Job plot of the mole fraction of the ligand against the relative absorption at 271.5 nm (**C**).

First, the complexation properties of the aroyl-*S*,*N*-ketene acetal **3b** with gold(I) iodide are investigated (Fig. 3). This initially revealed an interesting effect when looking at the emission properties during complexation: there is a continuous increase in the emission intensity and an associated increase from *Φ*_*f*_ = 0.20 in the absence of a metal salt to *Φ*_*f*_ = 0.55 in presence of AuI (2.43 equivs) (Fig. 3B). A further increase of the amount of metal salt causes a slight decrease in the emission intensity.

The formation of difference spectra before and after addition of the metal salt reveals the most pronounced change for the absorption maximum at a wavelength of 271.5 nm (Fig. 3A). The corresponding absorbance values of the difference spectra at this maximum are plotted against the corresponding mole fraction χ of the ligand gives the respective Job plot that is fitted by a quadratic correlation with *f*(χ) =  − 10.308 − χ^2^ + 10.190 − χ − 2.129 and an excellent correlation factor (R^2^ = 0.96). The paraboloid course of the function and the maximum of the negative parabola is localized at a mole fraction of χ = 0.5 indicates a weakly coordinating complex of aroyl-*S*,*N*-ketene acetal **3b** and Au^+^ ion. (Fig. 3C).

A similar behavior could be observed for both iron and ruthenium salts. Since the Fe^3+^-complexing properties of a benzothiazole croconaine derivative^64^ served as the motivation for investigating the complexing properties of aroyl-*S*,*N*-ketene acetals, aroyl-*S*,*N*-ketene acetals are intriguing to be also checked for complexation with Fe^3+^ ions. Iron(III) chloride is used as the metal salt for this purpose, and complexing properties of the aroyl-*S*,*N*-ketene acetals are indeed revealed. Once again, there is a clear increase in fluorescence intensity due to complexation Fe^3+^ ions to *Φ*_*f*_ = 0.59 (see SI, Fig. S9B) and for Ru^3+^ ions to 0.57 (see SI, Fig. S10B). Both, iron (*f*(χ) =  − 12.646 − χ^2^ + 10.991 − χ − 1.152) and ruthenium (*f*(χ) =  − 0.499 − χ^2^ + 0.597 − χ − 1.521) show good correlations with their respective fits of 0.93 and 0.86. For Fe^3+^-ions, a weakly coordinating 1:1 complex can be observed (see SI Fig. S9) while for Ru^3+^-ions, a weak complex with a 1:2 stoichiometry seems to be rational (see SI Fig. S10).

Several possible explanations for the observed fluorescence enhancements upon ligation can be given. The enhancement may result from ion-induced changes in the geometry or flexibility of the ligand. Other possibilities include chelation-induced changes of the relative energetic positions of different states, which shut off deactivation pathways as well as surpassing radiationless deactivation pathways via torsional motions which may be a viable explanation for aroyl-*S*,*N*-ketene acetals. The determination of the mechanism behind these fluorescence enhancements have been studied for more than 20 years and are still basis of currently ongoing investigations^69–71^.

Theoretically, various complexation modes of the dimethylamino-aroyl-*S*,*N*-ketene acetal **3b** are conceivable. Coordination can either take place via the lone pairs of the dimethylamino group, the carbonyl oxygen, the sulfur atom of the benzothiazole or simultaneously via the carbonyl oxygen and the sulfur atom, i.e. by chelation. Previous studies suggested coordination via carbonyl oxygen and benzothiazole sulfur for 1:1 stoichiometry of comparable ligand systems^64^. In the case of the ruthenium complex, it can therefore be assumed that the ruthenium can be coordinated via carbonyl oxygen and benzothiazole sulfur as well as via the dimethylamino unit^72–74^.

For bichromophore system **5a**, a pronounced effect of fluorescence quenching can be observed with the addition of iron(III) chloride; a weak effect can also be observed with regard to absorption spectroscopy.

**Figure 4:**
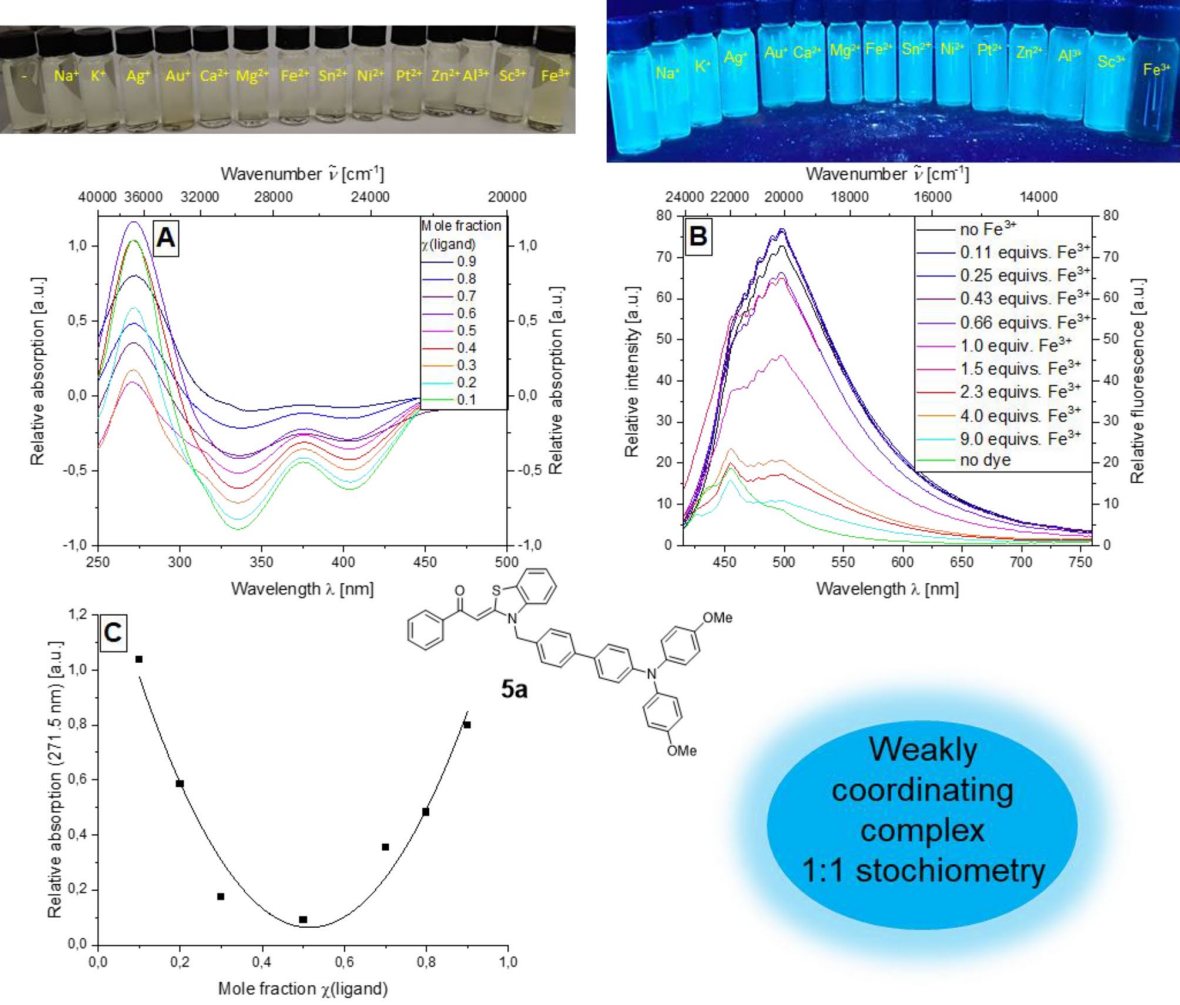
Top: apparent perception of the properties of **5a** when different metal salts are added in daylight (left) and under UV light (right); middle: absorption difference spectra of compound **5a** at different mole fractions of the ligand dye in the presence of FeCl_3_ (recorded in ethanol; *c*(**5a**) = 10^−5^m, *c*(FeCl_3_) = 10^−5^ m, T = 298 K) (**A**), Emission spectra of compound **5a** with increasing amount of FeCl_3_ (*c*(**5a**) = 10^−7^ m, *c*(FeCl_3_) = 10^−7^ m, T = 298 K, *λ*_*exc*_ = 373 nm) (**B**); bottom: Job plot of the mole fraction of the ligand against the relative absorption at 271.5 nm (**C**).

In contrast to the complexometric studies of **3b** (Fig. 4A), a decrease in fluorescence intensity of the bichromophore **5a** can be observed with increasing mole fraction until the emission is finally extinguished (Fig. 4B). One possibility for the fluorescence quenching are competing electron transfer processes: the ligand is excited and Fe(III) is thus reduced to Fe(II) by the excited ligand. Another possible explanation addresses the effect of the comparable quenching behavior during protonation which we reported earlier for triphenylamine bichromophores^57^. The fluorescence can be attributed to the emissive triphenylamine secondary chromophore. However, an energy transfer to the aroyl-*S*,*N*-ketene acetal system can be assumed^57^. The systemic change in the molecule takes place at the aroyl-*S*,*N*-ketene acetal motif, which is responsible for the fluorescence quenching, and not at the triphenylamine, which is affected neither by complexation nor protonation.

The most obvious assumption of the complexation site is the sulfur and oxygen atoms of the aroyl unit and the benzothiazole unit.

The determination of the difference spectra showed that the most pronounced effect could be observed for the absorption maximum at 271.5 nm. Therefore, this maximum was selected for the creation of the corresponding Job plot.

Plotting the mole fraction of the ligand against the relative absorbance at 271.5 nm results in a paraboloid curve, which indicates a weakly coordinating complex. The position of the maximum at χ = 0.5 indicates a 1:1 stoichiometry of the fluorescence quenching complex (Fig. 4C).

**Figure 5:**
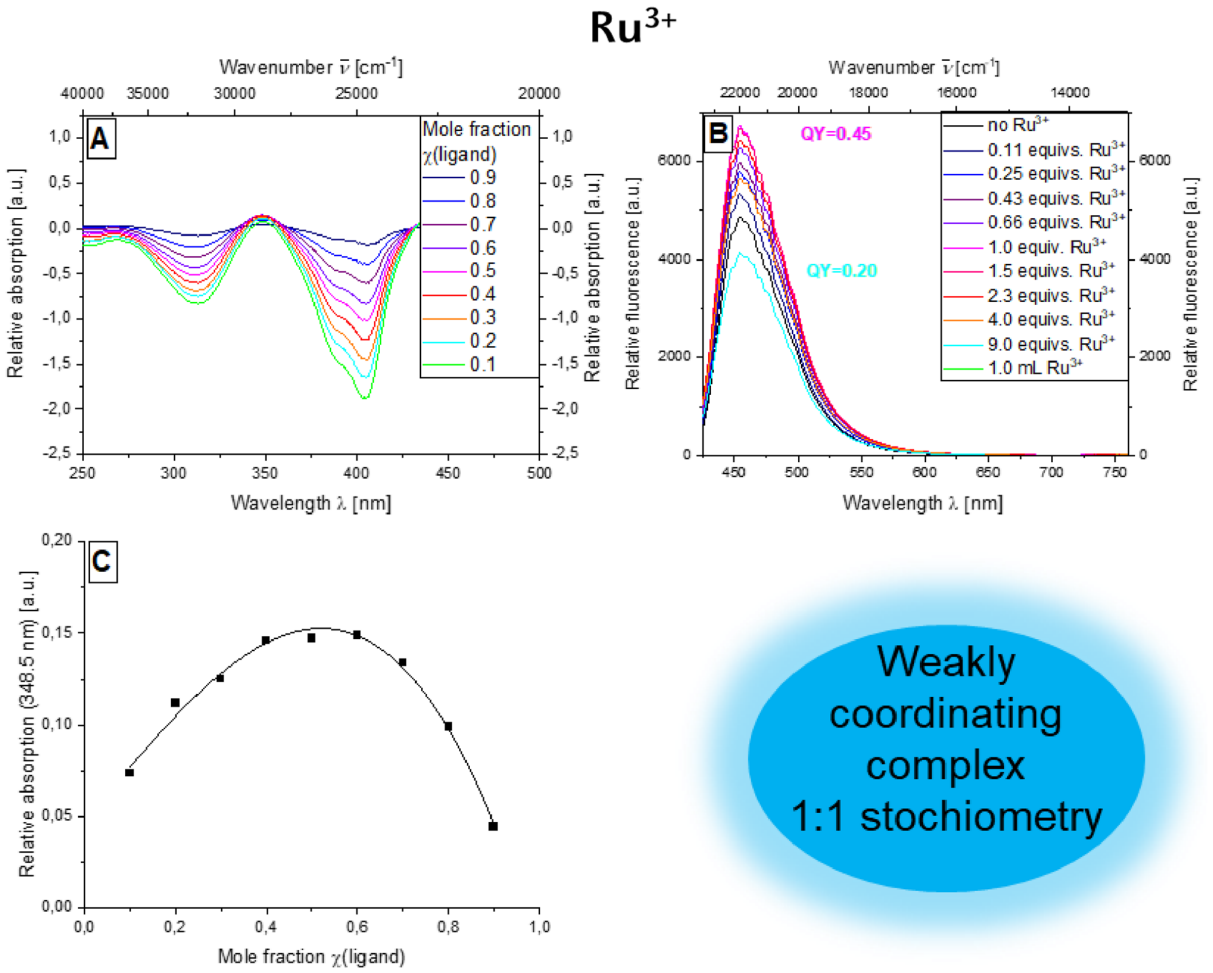
Absorption difference spectra of compound **5b** at different mole fractions of the ligand dye in the presence of RuCl_3_ (recorded in ethanol; *c*(**5b**) = 10^−5^ m, *c*(RuCl_3_) = 10^−5^ m, T = 298 K) (**A**), emission spectra of compound **5b** with increasing amount of RuCl_3_ (*c*(**5b**) = 10^−7^ m, *c*(RuCl_3_) = 10^−7^ m, T = 298 K, *λ*_*exc*_ = 404 nm) (**B**), Job plot of the mole fraction of the ligand against the relative absorption at 348.5 nm (**C**).

As for compound **3b**, it was also possible to proceed for the combination of ruthenium(III) chloride and bichromophore **5b**. With regard to the emission intensity, the intensity can also be increased by adding the dissolved metal salt. At the same time, the fluorescence quantum yield can be more than doubled from *Φ*_*f*_ = 0.20 to 0.45 (Fig. 5B). In the case of ruthenium(III) chloride, the difference spectra reveal that the absorption maximum at 348.5 nm is most suitable for investigation using Job plot analysis (Fig. 5A). The Job plot of the mole fraction of the ligand χ against the relative absorbance at 348.5 nm provides a parabola that is highly correlated with the individual measurement points, so that a weakly coordinating complex can also be detected in this case. The maximum of this parabola was at χ = 0.5, meaning that a 1:1 stoichiometry of the ruthenium dye complex can be assumed (Fig. 5C).

Although no complex formation can be observed for other metal ions, the presence of various metal ions nevertheless has an obvious influence on the absorption and emission properties of bichromophore **5b** dissolved in ethanol during qualitative tests. For this purpose, low concentrations of both the bichromophore **5b** and the respective metal salt of *c* = 10^−5^ m for absorption and *c* = 10^−7^ m for emission are used. While an absorption maximum at 315 nm can be observed in the absence of a metal salt, this maximum disappears in the presence of metal ions. For the presence of gold ions, the maximum mentioned appears at 271.5 nm. The addition of ruthenium(III) chloride causes a bathochromic shift of the long wavelength absorption maximum. Furthermore, the addition of iron(II) sulfate, iron(III) chloride and aluminum(III) chloride results in a longer wavelength absorption maximum which can be ascribed to the long wavelength metal to ligand charge transfer bands. This absorption maximum occurs for Fe^2+^ ions at 495 nm, for trivalent iron ions this maximum occurs with increased intensity bathochromically shifted to 505 nm.

**Figure 6:**
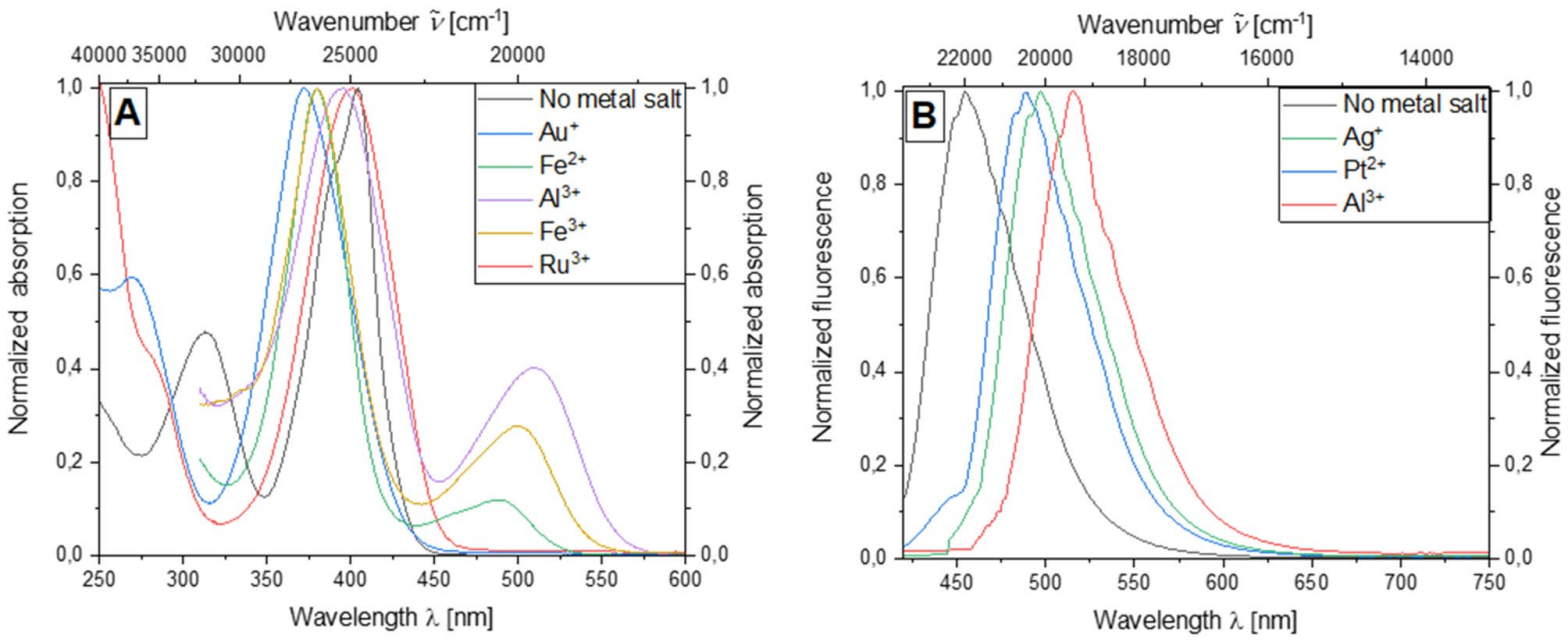
Absorption spectra on addition of various metal salts in ethanol (*c*(**5b**) = 10^−5^ m, *c*(M^+^) = 10^–5^ m, T = 298 K) (**A**) and emission spectra of compound **5b** on addition of various metal salts in ethanol (*c*(**5b**) = 10^−7^ m, *c*(M^+^) = 10^−7^ m, T = 298 K, *λ*_*exc*_ = 404 nm).

This effect can be observed even more strongly in the presence of Al^3+^ ions, where the maximum occurs at 513 nm (Fig. 6A). With regard to the emission, an effect due to the presence of metal ions can also be demonstrated. Without the addition of metal salts, the emission maximum occurs at 447 nm. In presence of Pt^2+^ ions and Ag^+^ ions, a bathochromic shift of the emission maximum to 497 and 502 nm, respectively, can be observed. The most significant effect occurred in the presence of Al^3+^ ions. The respective emission maximum can be detected here at 520 nm (Fig. 6B).

In comparison to the other systems **3b**, **5a**, and **5b** investigated, a strongly altered emission behavior of the bis(dimethylamino-aroyl-*S*,*N*-keteneacetal)-1,10-phenanthroline system **5c** can be observed. So far, the presence of the metal salts has led to a significant increase in the fluorescence quantum yield *Φ*_*f*_. In the case of the presence of the phenanthroline linker, the pronounced fluorescence quantum yield of the dimethylamino-aroyl-*S*,*N*-ketene acetals is already significantly reduced to 0.04. In addition, the fluorescence quantum yield *Φ*_*f*_ decreases significantly with increasing mole fraction of the gold(I) iodide, with the greatest drop in emissivity already occurring upon addition of 0.11 equivs of gold(I) iodide. The further decrease in the fluorescence quantum yield *Φ*_*f*_ with increasing mole fraction is almost linear (see SI, Fig. S15B).

**Figure 7:**
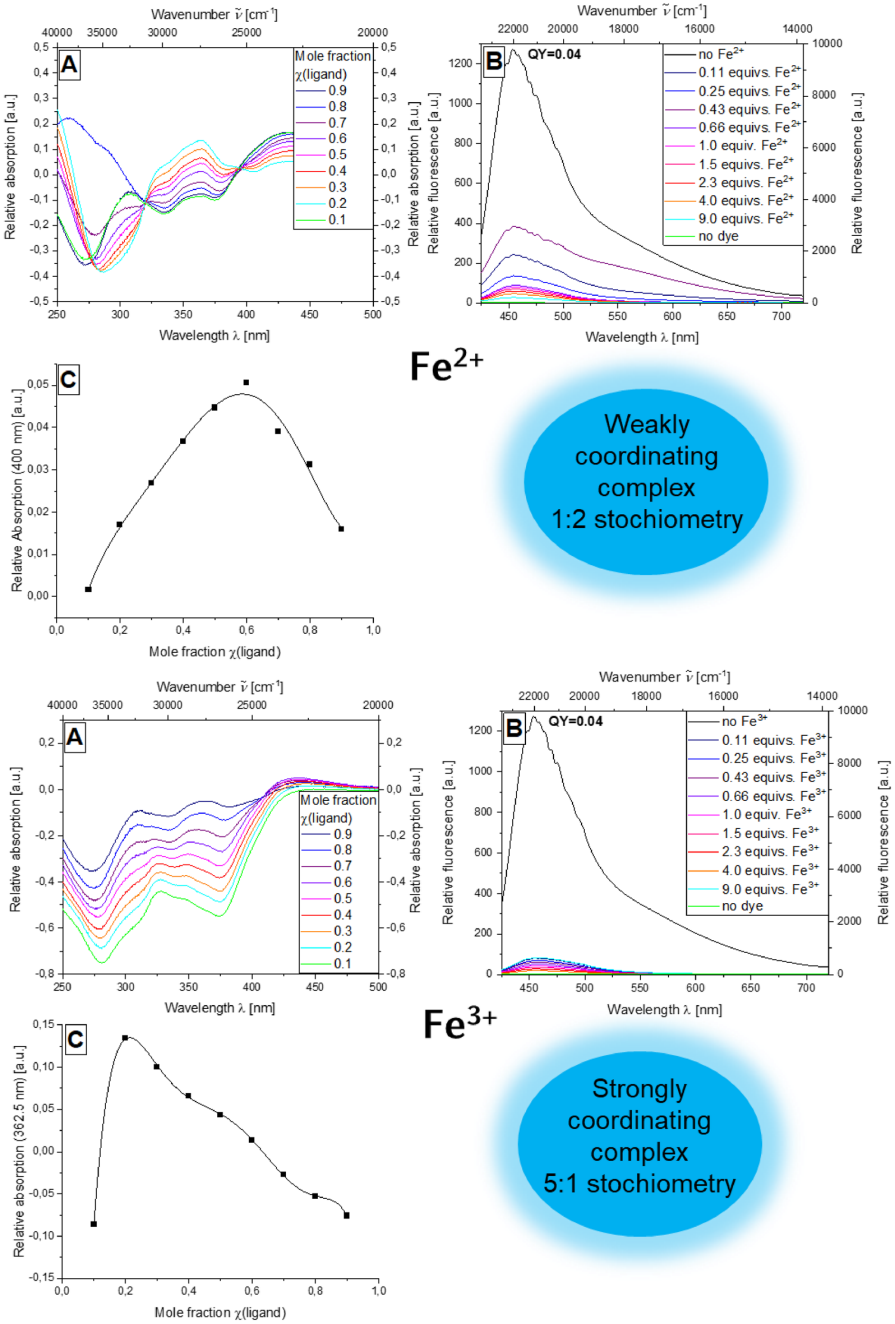
Absorption difference spectra of compound **5c** at different mole fractions of the ligand dye in the presence of FeSO_4_ (top) and FeCl_3_ (bottom) (recorded in ethanol; *c*(**5c**) = 10^−5^ m, *c*(metal salt) = 10^−5^ m, T = 298 K) (**A**), emission spectra of compound **5c** with increasing amount FeSO_4_ (top) and FeCl_3_ (bottom) (*c*(**5c**) = 10^−7^ m, *c*(metal salt) = 10^−7^ m, T = 298 K, *λ*_*exc*_ = 404 nm) (**B**), Job plot of the mole fraction of the ligand against the relative absorption at 271.5 nm (FeSO_4_, top) and (FeCl_3_, bottom) (**C**).

The formation of the corresponding difference spectra reveals the greatest change for an absorption wavelength of 449 nm, so that these respective absorption values of the difference spectra at different mole fractions of the ligand could be used for the analysis using a Job plot (Fig. 7, top A). It is possible to fit the course of the complexation behavior by a quadratic function, so that formation of a weakly coordinating complex can be assumed. The maximum of the parabola is localized at a mole fraction of χ = 0.65, so that a complex consisting of two Au^+^ ions and a molecule of the 1,10-phenanthroline system **5c** is formed (see SI, Fig. S15C).

In contrast to previously investigated systems, complexation of Fe^2+^ ions is observed in the case of derivative **5c**, such as complexation must be attributable to the 1,10-phenanthroline linker. It can be seen that quenching of the emission in the presence of the metal salt is a material property of derivative **5c**, since the initial fluorescence quantum yield *Φ*_*f*_ of 0.04 decreases significantly when the metal salt loading is increased (Fig. 7, top B).The difference spectra of compound **5c** reveal that the absorbance maximum at 400 nm is ideal for examination by Job plot analysis (Fig. 7, top right A). The plot of the absorbance at 400 nm against the mole fraction χ of the ligand again shows an almost parabolic curve and thus indicating a weakly coordinating complex. The position of the maximum at 0.6 suggests the formation of an iron-dye complex in a 1:2 stoichiometry (Fig. 7, top C).

Finally, the complexation behavior of the multichromophore **5c** with respect to iron(III) chloride (Fig. 7, bottom A) will be discussed. The quenching of the emission reaches its maximum with this metal salt and chromophore combination. After addition of 0.11 equivs of iron(III) chloride, almost complete quenching of the emission intensity is observed. The fluorescence quantum yield *Φ*_*f*_ decreases from 0.04 without any metal salt present to well below 0.01 upon the addition of the respective metal salt (Fig. 7, bottom B). With regard to the difference spectra obtained, the local maximum can be identified as suitable for Job plot analysis (Fig. 7, bottom A).The job plot of the mole fraction of the ligand χ against the relative absorbance at 362.5 nm yields a 7th degree function that is highly correlated with the individual measurement points and consists of two almost linear subsections, so that a strongly coordinating complex can be assumed in this case. At the same time, the position of the maximum of the 7th degree function at χ = 0.2 indicates the formation of a complex with a 5:1 stoichiometry consisting of five ligands and one Fe^3+^ ion^67,68^. This behavior can be interpreted that Fe^3+^ ions coordinate to both 1,10-phenanthroline and the dimethylamino aroyl-*S*,*N*-ketene acetals (Fig. 7, bottom C).

**Figure 8:**
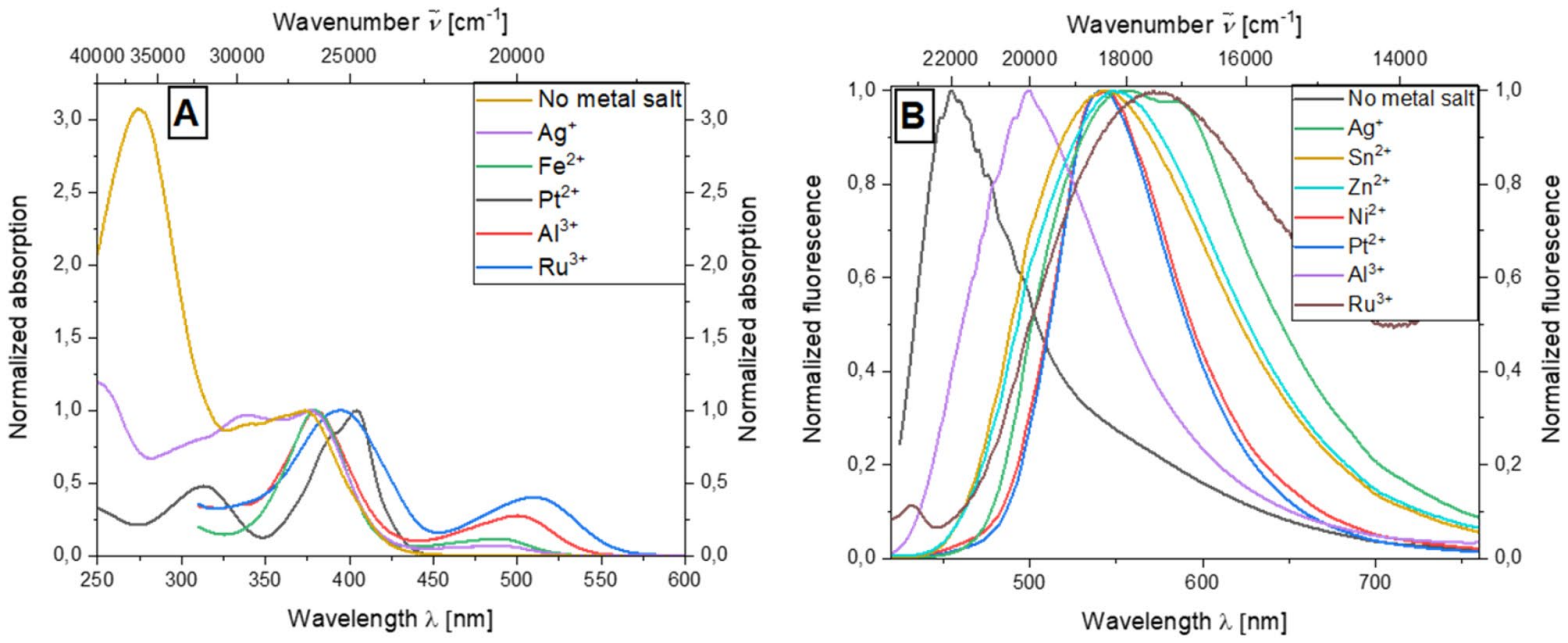
Absorption spectra on addition of various metal salts in ethanol (*c*(**5c**) = 10^−5^ m, *c*(M^+^) = 10^−5^ m, T = 298 K) (**A**) and emission spectra of compound **5c** on addition of various metal salts in ethanol (*c*(**5c**) = 10^−7^ m, *c*(M^+^) = 10^−7^ m, T = 298 K, *λ*_*exc*_ = 404 nm).

Even if no stable and observable metal dye complexes are formed, the presence of metal ions still have a considerable effect on both absorption and emission properties of dye **5c**. To quantify the influence of the presence of the metal ions, low concentrations of *c* = 10^−5^ m of both the metal salt used and the bridged system **5c** were used for emission and concentrations of *c* = 10^−7^ m were used. The intense absorption maximum at 274 nm disappears almost completely in the presence of various metal salts. For some metal salts, the absorption at 320 nm is only plotted, as interfering absorption effects of the metal salt solutions occur at higher energies. For platinum(II) salts and silver(I) iodide, the formation of an additional, energetically higher absorption maximum can be detected at 318 and 341 nm, respectively. The absorption maximum which can be ascribed to the aroyl-*S*,*N*-ketene acetal unit occurring at 373 nm in the absence of the metal salts occurs almost unchanged in the presence of Ag^+^, Fe^2+^ and Al^3+^ ions (Fig. 8A).

A similarly distinct behavior can also be detected for the emission of system **5c** in the presence of various metal salts. In the absence of additives, the emission maximum in ethanol occurs at 455 nm. The emission is bathochromically shifted to 498 nm in the presence of Al^3+^ ions, resulting in a greenish fluorescence. For the presence of Ni^2+^ and Pt^2+^ ions, the emission maximum in solution can be observed at 531 nm, whereas the emission intensity is clearly different and by a factor of 10 lower for the presence of Ni^2+^ ions. Comparably, the emission maxima occur with the addition of Sn^2+^ and Zn^2+^ ions at 542 and 546 nm, respectively, with significantly broadened emission bands. The solution of chromophore **5c** luminesces most intensely in the presence of silver(I) ions. Two emission maxima can be observed here, at 549 and 602 nm. The presence of Ru^3+^ ions results in the most pronounced bathochromic shift of the emission maximum. The solution of the dye luminesces orange in ethanol with an emission maximum of 592 nm (Fig. 8B).

**Figure 9:**
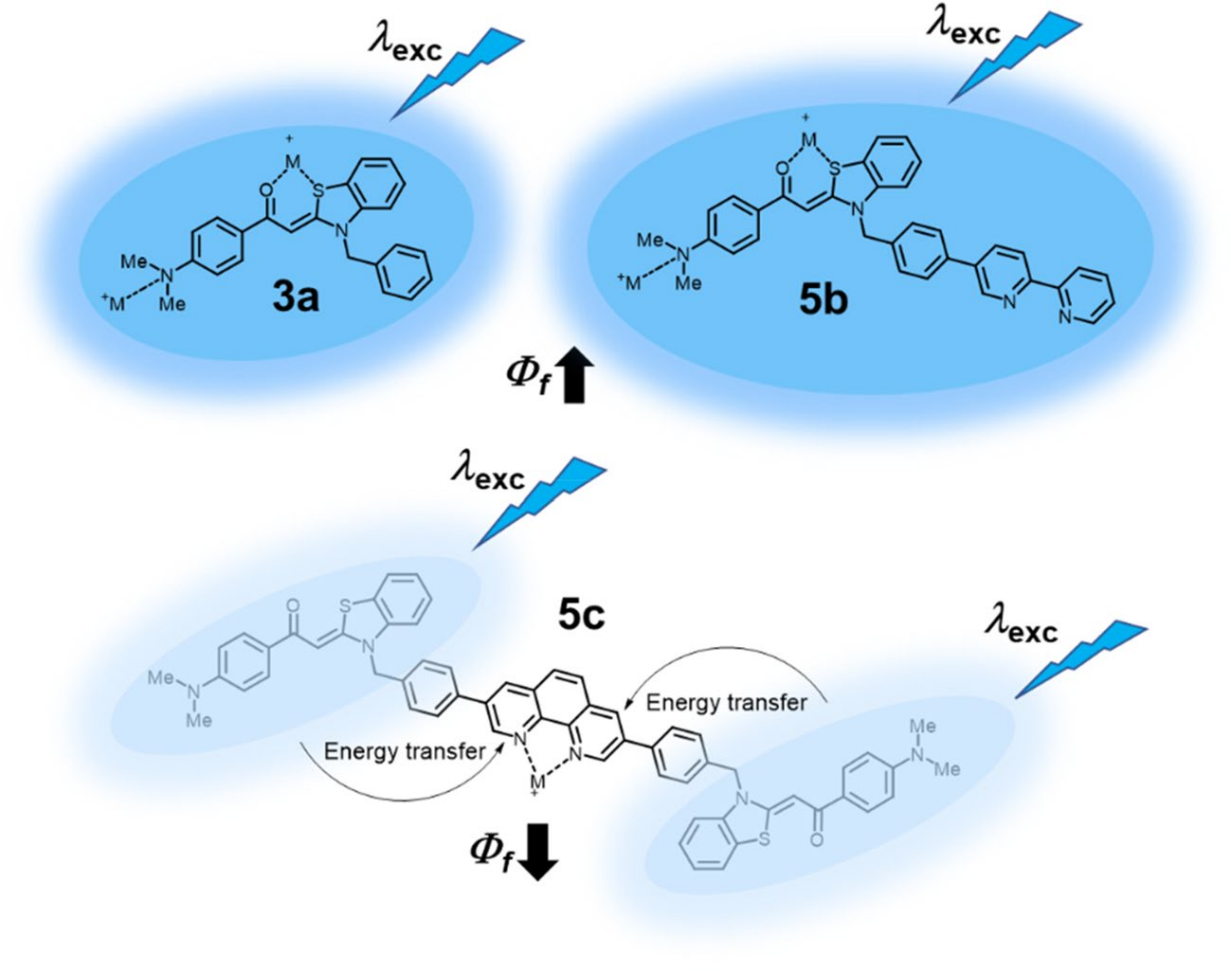
Potential explanatory approach to rationalize the emission enhancement of **3a** and **5a** and the quenching of the emission of **5c** in the presence of metal ions.

The discrepancy between the emission behavior of the derivatives **3a** and **5b** and the derivative **5c** and their spectroscopically detected metal complexes suggests that two fundamentally divergent binding modes of the metal complexes are operative. It can be concluded that for chromophores **3a** and **5b** a constructive influence of the metal ions is in effect as emission quenching by energy or electron transfer does not occur in these cases. The most likely sites of coordination are in this case either the dimethylamino functionality or the potentially chelating oxygen-sulfur unit of aroyl and benzothiazole. For dimethylamino aroyl-*S*,*N*-ketene acetals, it is reasonable to assume that complexation also occurs via the amino nitrogen. But, as with all anilines, both the Brønsted and Lewis basicity is reduced by the aromatic compound, and even more so by the p-conjugation to the carbonyl group. Therefore, the carbonyl group even becomes more electron-rich as a result. Ergo, chelation, also for thermodynamic reasons, enthalpic and entropic, should actually be the most likely modus operandi for these systems. This means that the presence of the metal salt can have a fluorogenic effect as seen by the increase in the fluorescence quantum yield *Φ*_*f*_ (Fig. 9, top)^23,75–77^_._ A possible explanation for this behavior lies in the metal ion-induced changes in the geometry and flexibility of the ligand as well as reducing the accessibility of deactivation pathways of certain functional groups. Especially in case of flexible ligands like bipyridine or equally flexible aroyl-*S*,*N*-ketene acetal, the chelation process suppresses radiationless deactivation via torsional motions^40^.

The quenching of the emission in the presence of transition metal ions, as observed for **5c**, clearly indicates that energy or electron transfer processes, as described in the literature^78,79^, lead to emission quenching. Decisive for such processes the corresponding metal must coordinate to the central 1,10-phenanthroline, which has been shown to act as a quencher^80,81^. Therefore, it can be assumed that a quenching energy transfer (EnT) process from the emissive dimethylamino aroyl-*S*,*N*-ketene acetals to the dark, non-emissive phenanthroline-metal complex can occur (Fig. 9, bottom)^76,77^.

## Conclusion

In summary, bi-, multi- and single aroyl-*S*,*N*-ketene acetals were readily synthesized in mostly very good to excellent yields. Aside from typical AIE properties, these dyes exhibit pronounced complexometric behavior. Ruthenium, iron and gold cations form complexes with the investigated dyes as seen by photospectrometry. For most of the compounds, the dimethylamino substituent or the carbonyl oxygen and the sulfur of the benzothiazole are most likely sites of complexation resulting in a significant increase of quantum yield. In case of dimethylamino aroyl-*S*,*N*-ketene acetals and Au^+^-ions, the fluorescence quantum yield is nearly tripled upon complexation. Similar trends were also observed for iron and ruthenium ions and in case of dimethylamino aroyl-*S*,*N*-ketene acetal bipyridine bichromophores. In contrast, for the 1,10-phenanthroline bridged trichromophore, the quantum yield drops presumably due to a dark state of the complexed 1,10-phenanthroline unit. For most metal ion-dye combinations, only weakly coordinating complexes could be observed by spectroscopy, only for a phenanthroline aroyl-*S*,*N*-ketene acetal multichromophore in combination with Fe^3+^-ions, the formation of a strongly coordinating complex could be observed. In addition, even if no stable complexes are formed, the change of the emission color in the presence of different metal ions can be exploited for metal ion sensing. Future works are directed to address metal complexation for ion sensing and fishing, as well as to form more stable complexes.

## Methods

The used chemicals which have not been synthesized were purchased at Acros Organics BVBA, Alfa Aeser GmbH & Co KG, Fluorochem Ltd., J&K Scientific Ltd., Merck KGaA, Macherey–Nagel GmbH & Co. KG, Sigma-Aldrich Chemie GmbH and VWR and have been used without further purification. The melting points have been measured with Melting Point B-540 of the company Büchi according to the protocol by *Kofler*^82^. All NMR and mass spectrometry experiments have been performed by the Heinrich Heine University Center of Molecular Structure Analytics (HHUCeMSA). ^1^H, ^13^C and DEPT 135-spectra have been measured at 298 K on an Avance III—300 and an Avance III—600 of the company Bruker. EI mass spectra have been measured with Triple Quadrupol spectrometer TSQ 7000 of the company Finnigan MAT. MALDI spectra have been measured with a MALDI/TOF UltrafleXtreme of the company Bruker Daltronik. IR spectra were recorded with neat compounds under attenuated total reflection (ATR) with IRAffinity-1 of the company Shimadzu. The elementary analyses have been measured with Perkin Elmer Series II Analyser 2400 or Vario Micro Cube of the company Analysensysteme GmbH. UV/Vis spectra of the dye solutions were measured with a Lambda 19 spectrometer from Perkin Elmer. The emission spectra of the dye solutions and the solid compounds were recorded with a Hitachi F-7000 spectrofluorometer using the emission correction curve provided by the instrument manufacturer. Emission spectra were not corrected for the wavelength-dependent spectral responsivity of the fluorometer. All solution spectra were recorded with dyes dissolved in spectroscopic grade solvents at 298 K using 1 cm-_quat_z cuvettes from Hellma GmbH. The molar extinction coefficients of dye solutions of known dye concentration were determined by five-point regression line.

For aggregation studies, samples of the aroyl-*S*,*N*-ketene acetals were dissolved in various mixtures of organic solvents and water with water contents ranging from 0 to 99%, with ethanol/water mixtures giving the clearest results, which are discussed in detail below. Furthermore, solvent mixtures of cyclohexane and dichloromethane were also tested, but no aggregate formation was observed. A stock solution of the respective chromophore was prepared in ethanol. A defined amount of this stock solution was taken, and the corresponding amount of ethanol was first placed in a 10 mL volumetric flask before the corresponding amount of water was added. To exclude aging and coagulation processes of the formed aggregates as far as possible, all solutions were first treated in an ultrasonic bath for 5 min before the emission and absorption spectra were recorded starting with the solutions of the highest water content. Selected aggregate solutions were stored for investigation of aggregate stability over time for subsequent measurements at defined time points. For the photographs of the aggregate solutions, the corresponding measurement solutions were used directly.

### Supplementary Information

Supplementary Information.

## Data Availability

All data generated or analyzed during this study are included in this published article and its supplementary information files. Experimental details of all synthesized derivatives and the respective spectroscopic and analytic data, spectra (^1^H and ^13^C NMR spectra, absorption and emission spectra, titration experiments) and photographs, see the accompanying Supporting Information. The datasets used and/or analyzed during the current study are available from the corresponding author on reasonable request.
