# Supplementary material for: The complexometric behavior of selected aroyl-S,N-ketene acetals shows that they are more than AIEgens
Source: Sci Rep. 2024 May 31;14:12565. doi: 10.1038/s41598-024-62100-4 (PMC11143253; doi:10.1038/s41598-024-62100-4)
Supplement: Supplementary file 1 — Supplementary Information. [file 41598_2024_62100_MOESM1_ESM.pdf]

The Complexometric Behavior of Selected Aroyl-*S*,*N*-Ketene Acetals Shows that They Are More than AIEgens

Lukas Biesen,[1,2] and Thomas J. J. Müller[1,*]

[1] Heinrich-Heine-Universität Düsseldorf, Math.-Nat. Fakultät, Institut für Organische Chemie und Makromolekulare Chemie, Universitätsstraße 1, D-40225 Düsseldorf, Germany E-Mail: ThomasJJ.Mueller@uni-duesseldorf.de

[2] New address: School of Chemistry, Joseph Black Building, University of Glasgow, G12 8QQ, United Kingdom

1 General considerations 2
2 Overview of synthesized ligands 4
3 Starting material 5
4 Synthesis and analytical data of aroyl-*S*,*N*-ketene acetals **3** 7
4.1 General procedure (GPI) for the synthesis of aroyl-*S*,*N*-ketene acetals **3**........ 7
4.2 Synthesis and analytical data of secondary chromophores **4** 12
4.2.1 4-Bromo-*N*,*N*-bis(4-methoxyphenyl)aniline........12
4.2.2 4-Methoxy-*N*-(4-methoxyphenyl)-*N*-(4-(4,4,5,5-tetramethyl-1,3,2-dioxaborolan-2-yl)phenyl)aniline (**4a**)........13
4.2.3 5-(4,4,5,5-Tetramethyl-1,3,2-dioxaborolan-2-yl)-2,2'-bipyridine (**4b**) 14
4.3 Synthesis and analytical data of bi- and multichromophores **5** 16
4.3.1 (*Z*)-2-(3-((4'-(Bis(4-methoxyphenyl)amino)-[1,1'-biphenyl]-4-yl)methyl)-benzo[*d*]thiazol-2(3*H*)-ylidene)-1-phenylethan-1-one (**5a**) 16
4.3.2 (*Z*)-2-(3-(4-([2,2'-Bipyridine]-5-yl)benzyl)benzo[*d*]thiazol-2(3*H*)-ylidene)-1-(4-(dimethylamino)phenyl)ethan-1-one (**5b**) 18
4.3.3 (2*Z*,2'*Z*)-2,2'-((((1,10-Phenanthrolin-3,8-diyl)bis(4,1-phenylene))bis(methylene))bis(benzo[*d*]thiazol-3(3*H*)-yl-2(3*H*)-ylidene))bis(1-(4-(dimethylamino)phenyl)ethan-1-one) (**5c**) 20
5 NMR spectra 22
6 Overview of photophysical properties of aroyl-*S*,*N*-ketene acetal metal sensors **3** and **5**........28
7 Absorption and emission spectra 29
7.1 Absorption and emission spectra of aroyl-*S*,*N*-ketene acetal metal sensors........29
8 Complexometry........35

1 General considerations

Reactions were carried out in dried and sintered Schlenk tubes or round bottom flasks under nitrogen atmosphere. Solvents were dried by a solvent purification system *MB-SPS-800* of the company *MBraun Inertgas-Systeme GmbH*.
The used chemicals, which have not been synthesized, were purchased at *Acros Organics BVBA, Alfa Aeser GmbH & Co KG, Fluorochem Ltd., J&K Scientific Ltd., Merck KGaA, Macherey-Nagel GmbH & Co. KG, Sigma-Aldrich Chemie GmbH* and *VWR* and have been used without further purification. The solvents ethanol and tetrahydrofuran (THF) (spectroscopy grade) were purchased from *Merck* and *Sigma-Aldrich* (Germany), respectively. Milli-Q-water was obtained from a *Millipore water purification system*. Carboxylated 8 µm-sized polystyrene particles (PSP) were obtained from *Kisker Biotech GmbH* (Germany).

Further purification of the compounds was achieved by flash column chromatography (silica gel M60 pore size 0.040-0.063 nm) of the company *Macherey-Nagel*. The crude product was adsorbed on Celite®545 of the company *Carl Roth GmbH*, placed on the suspended silica gel and purified with a positive pressure of 2 bar. Distilled solvent mixtures of *n*-hexane, acetone and methanol have been used as eluents.

The control of reaction progress was done via thin layer chromatography (TLC) with silica coated aluminium plates F_{254}, of the company *Macherey-Nagel GmbH & Co. KG.*

The melting points have been measured with *Melting Point B-540* of the company *Büchi* according to the protocol by *Kofler*.

All NMR and mass spectrometry experiments have been performed by the Heinrich Heine University Center of Molecular Structure Analytics (HHUCeMSA). ^{1}H,^{13}C and DEPT 135-spectra have been measured at 298 K on an *Avance III - 300* and an *Avance III - 600* of the company *Bruker*. Chemical shifts in the ^{1}H and ^{13}C NMR are reported in ppm relative to deuterated solvents such as acetone-d_6 (δ_H 2.05, δ_C 29.84, δ_C 206.26) with CS_2 (δ_C 192.28) and DMSO-d_6 (δ_H 2.50, δ_C 39.51). The multiplicity is abbreviated as follows: s = singlet; d = doublet; t = triplet; td = triplet of doublet; dd = doublet of doublet; dt = doublet of triplet, dq = doublet of quartet; pd = quintet of doublet m = multiplet. The assignment of primary carbon centers (CH), secondary carbon centers (CH_2), tertiary carbon centers (CH_3) and quaternary carbon centers (C_{quat}) were made by using DEPT-135 spectra.
EI mass spectra have been measured with Triple-Quadrupol-spectrometer *TSQ 7000* of the company *Finnigan MAT*. MALDI spectra have been measured with a *MALDI/TOF UltrafleXtreme* of the company *Bruker Daltronik*.

IR spectra were recorded with neat compounds under attenuated total reflection (ATR) with *IRAffinity-1* of the company *Shimadzu* and the intensities were characterized as strong (s), middle (m) and weak (w).

The elementary analyses have been measured with *Perkin Elmer Series II Analyser 2400* or *Vario Micro Cube* of the company *Analysensysteme GmbH* at the microanalytical laboratory of the institute for Pharmaceutical and Medicinal Chemistry of the University Düsseldorf.

UV/Vis spectra of the dye solutions were measured with a *Lambda 19* spectrometer from *Perkin Elmer*. The emission spectra of the dye solutions and the solid compounds were recorded with a *Hitachi F-7000* spectrofluorometer using the emission correction curve provided by the instrument manufacturer. All solution spectra were recorded with dyes dissolved in spectroscopic grade solvents at 298 K using 1 cm-$_{quat}$z cuvettes from *Hellma GmbH*. The molar extinction coefficients of dye solutions of known dye concentration were determined by five-point regression line. The SC-30 integration sphere module of the *FS5 spectrofluorometer* from *Edinburgh Instruments* was used to determine the quantum yields, and the *FLUORACLE* software was used to evaluate the measurements using both the direct and direct & indirect methods. The measurements were taken with the *FS5 spectrofluorometer* from *Edinburgh Instruments* using the emission correction curve provided by the manufacturer.

2 Overview of synthesized ligands

Table S1: Overview of aroyl-*S*,*N*-ketene acetal ligands.

Entry	Aroyl-*S*,*N*-ketene acetal ligand (yield)
1	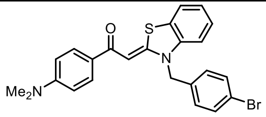 **3a** (317 mg, 65%)
2	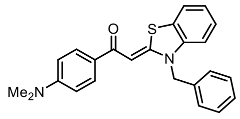 **3b** (278 mg, 52%)
3	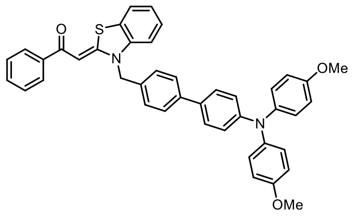 **5a** (214 mg, 66%)
4	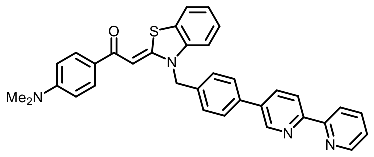 **5b** (157 mg, 97%)
5	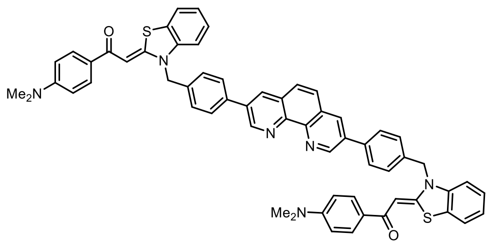 **5c** (116 mg, 49%)

3 Starting material

3-Benzyl-2-methylbenzo[*d*]thiazol-3-iumbromide (2a)

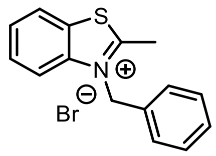

$C_{15}H_{14}BrNS$

[319.00]

2-Methylbenzothiazole (7.45 g, 50.0 mmol) and benzylbromide (10.2 g, 60.0 mmol) were placed in a round-bottom flask with a magnetic stir bar. The reaction mixture was stirred at 75 °C for 20 h, until the solution was completely hardened. After cooling to room temp the formed solid was filtrated via a Buechner funnel, washed with diethyl ether and dried under vacuo. The synthesis yielded 12.4 g (39.0 mmol, 78%) of the desired product **2a** as a pink solid.

Mp: 200 °C.

R_f (*n*-hexane/acetone 4:1): 0.10.

^{1}H NMR (300 MHz, DMSO-d$_6$): δ 3.30 (s, 3 H), 6.14 (s, 2 H), 7.33-7.40 (m, 5 H), 7.80 (pd, ^3J = 7.5 Hz, ^4J = 1.9 Hz, 2 H), 8.23 (dd, ^3J = 7.8 Hz, ^4J = 1.4 Hz, 1 H), 8.56 (dd, ^3J = 7.4 Hz, ^4J = 1.8 Hz, 1 H).

^{13}C NMR (75 MHz, DMSO-d$_6$): δ 17.5 (CH_3), 51.9 (CH_2), 117.1 (CH), 127.0 (CH), 128.3 (CH), 128.5 (CH), 129.1 (CH), 129.2 (C_{quat}), 129.5 (CH), 132.8 (C_{quat}), 140.8 (C_{quat}), 178.3 (C_{quat}).

EI + MS (70 eV, *m/z* (%)): 240 (15), 239 ($[C_{15}H_{14}NS]^+$, 67), 238 (65), 224 ($[C_{14}H_{10}NS]^+$, 14), 162 ($[C_9H_8NS]^+$, 13), 148 ($[C_8H_6NS]^+$, 32), 104 ($[C_7H_6N]^+$, 12), 91 ($[C_7H_7]^+$, 100), 65 ($[C_5H_5]^+$, 23).

3-(4-Bromobenzyl)-2-methylbenzo[*d*]thiazol-3-iumbromide (2b)

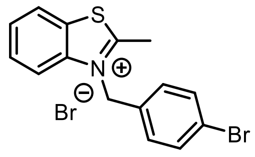

$C_{15}H_{13}Br_2NS$

[396.91]

2-Methylbenzothiazole (3.73 g, 25.0 mmol) and 4-bromo benzylbromide (5.10 g, 30.0 mmol) were placed in a round-bottom flask with a magnetic stir bar. The reaction mixture was stirred at 75 °C for 20 h, until the solution was completely hardened. After cooling to room temp the formed solid was filtrated via a Buechner funnel, washed with diethyl ether and dried under vacuo. The synthesis yielded 9.85 g (24.8 mmol, 99%) of the desired product **2b** as a pink solid.

Mp: 237 °C.

R_f (*n*-hexane/acetone 4:1): 0.10.

^{1}H NMR (300 MHz, DMSO-d$_6$): δ 3.25 (s, 3 H), 6.07 (s, 2 H), 7.29-7.32 (m, 2 H), 7.58-7.61 (m, 2 H), 7.82 (pd, ^3J = 7.5 Hz, ^4J = 1.9 Hz, 2 H), 8.18 (dd, ^3J = 7.5 Hz, ^4J = 1.5 Hz, 1 H), 8.50 (dd, ^1J = 7.4 Hz, ^2J = 1.8 Hz, 1 H).

^{13}C NMR (75 MHz, DMSO-d$_6$): δ 17.4 (CH_3), 51.3 (CH_2), 117.0 (CH), 121.8 (C_{quat}), 124.9 (CH), 128.2 (CH), 129.3 (CH), 129.4 (CH), 129.5 (C_{quat}), 131.9 (CH), 132.2 (C_{quat}), 140.9 (C_{quat}), 178.7 (C_{quat}).

EI + MS (70 eV, *m/z* (%)): 320 ($[C_{15}H_{13}{}^{81}BrNS^+$, 13), 319 ($[C_{15}H_{12}{}^{81}BrNS]^+$, 69), 318 ($[C_{15}H_{13}{}^{79}BrNS^+$, 57), 317 ($[C_{15}H_{12}{}^{79}BrNS]^+$, 68), 316 (51), 238 ($[C_{15}H_{13}NS]^+$, 13), 236 (11), 223 ($[C_{14}H_9NS]^+$, 12), 171 ($[C_7H_6{}^{81}Br]^+$, 95), 169 ($[C_7H_6{}^{79}Br]^+$, 100), 162 ($[C_9H_8NS]^+$, 23), 149 ($[C_8H_6NS]^+$, 15), 148 ($[C_8H_6NS]^+$, 34), 124 (13), 119 (18), 118 (21), 108 (12), 104 ($[C_7H_6N]^+$, 12), 90 ($[C_7H_6]^+$, 43), 89 (36), 82 (14), 63 (12).

4 Synthesis and analytical data of aroyl-*S,N*-ketene acetals 3

4.1 General procedure (GPI) for the synthesis of aroyl-*S,N*-ketene acetals 3

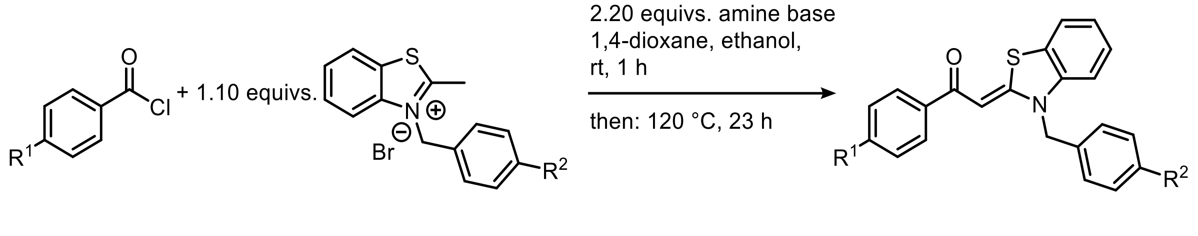

1 **2** **3**

Acid chloride **1** (1.00 equiv, 1.00 mmol) and 4 benzylbenzothiazoliumbromide **2** (1.10 equivs, 1.10 mmol) were placed in a sintered, dry screw-cap *Schlenk*-tube with a magnetic stir bar under nitrogen atmosphere and dissolved in 5 mL dry 1,4-dioxane and 2 mL ethanol. 2.20 equivs (2.20 mmol) amine base was added to the reaction mixture and the solution was stirred for 1 h at room temperature. Thereafter, the reaction mixture was stirred at 120 °C (oil bath) for 23 h. After cooling to room temp the crude product was absorbed onto Celite® and purified by flash chromatography on silica gel (*n*-hexane/acetone). The product was suspended in *n*-hexane, the sediment was filtrated and dried under vacuo.

Table S2: Experimental details for the synthesis of aroyl-*S,N*-ketene acetals **3**.

Entry	Acid chloride **1** [mg] ([mmol])	Benzylbenzo-thiazoliumbromide **2** [mg] ([mmol])	Yield of product **3** [mg] (%)
1[(a)]	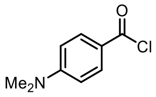 183 (1.00) of **1a**	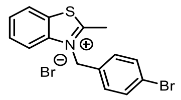 431 (1.10) of **2b**	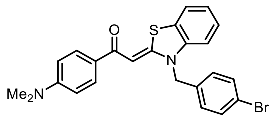 317 (68) of **3a**
2[(a)]	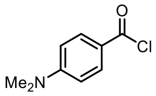 183 (1.00) of **1a**	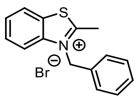 351 (1.10 mmol) of **2a**	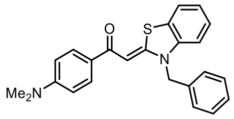 278 (72) of **3b**
3	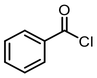 140 (1.00) of **1b**	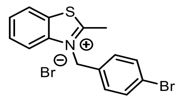 431 (1.10) of **2b**	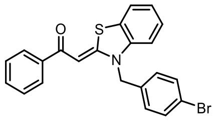 273 (65) of **3c**

(a): Diisopropylethylamine was used as a base instead of triethylamine and solely 1,4-dioxane as a solvent.

(*Z*)-2-(3-(4-Bromobenzyl)benzo[*d*]thiazol-2(3*H*)-ylidene)-1-(4-((dimethylamino)phenyl) ethan-1-one (3a)

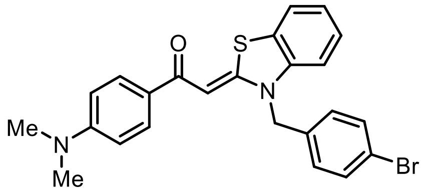

$C_{24}H_{21}BrN_2OS$

[464.06]

The synthesis was performed according to **GPI** to give 317 mg (0.683 mmol, 68%) of the desired product **3a** as a yellow solid.

Mp: 178 °C.

R_f (*n*-hexane/acetone 4:1): 0.11.

^{1}H NMR (300 MHz, acetone-d$_6$/CS$_2$ 5:1): δ 3.03, 3.06 (s, 6 H), 5.45 (s, 2 H), 6.62-6.75 (m, 4 H), 7.13-7.32 (m, 4 H), 7.51 (d, ^3J = 8.5 Hz, 1 H), 7.64 (d, ^3J = 7.6 Hz, 1 H), 7.76-7.79 (m, 3 H).

^{13}C NMR (75 MHz, acetone-d$_6$/CS$_2$ 5:1): δ 40.2 (CH_3), 40.4 (CH_3), 49.1 (CH_2), 88.0 (CH), 110.6 (CH), 111.8 (CH), 122.2 (CH), 129.96 (CH), 129.99 (CH), 123.4 (C_{quat}), 127.1 (CH), 128.5 (C_{quat}), 129.5 (C_{quat}), 129.6 (CH), 132.1 (CH), 132.8 (CH), 132.9 (CH), 135.6 (C_{quat}), 140.9 (C_{quat}), 153.0 (C_{quat}), 160.6 (C_{quat}), 183.7 (C_{quat}).

MALDI-TOF (*m/z*): 467.1 ($C_{24}H_{21}{}^{81}BrN_2OS+H^+$), 465.1 ($C_{24}H_{21}{}^{79}BrN_2OS+H^+$).

IR $\tilde{\nu}$ [cm^{-1}]: 658 (w), 689 (w), 698 (w), 712 (m), 745 (s), 768 (s), 799 (m), 822 (m), 881 (m), 945 (m), 970 (m), 1009 (m), 1047 (m), 1063 (m), 1088 (w), 1125 (m), 1163 (s), 1184 (s), 1242 (m), 1294 (m), 1319 (m), 1341 (m), 1371 (m), 1406 (m), 1443 (m), 1477 (s), 1533 (m), 1545 (s), 1578 (s), 1686 (w), 1734 (w), 2806 (w), 2889 (w), 2913 (w), 3049 (w), 3906 (w).

UV/Vis (C_3H_6O): λ_{max} (ε) = 404 (57700).

Anal calcd for $C_{24}H_{21}BrN_2OS$ [464.1]: C 61.94, H 4.55, N 6.02, S 6.89; Found: C 62.29, H 4.82, N 6.23, S 6.64.

(*Z*)-2-(3-Benzyl)benzo[*d*]thiazol-2(3*H*)-ylidene)-1-(4-((dimethylamino)phenyl)ethan-1-one (3b)

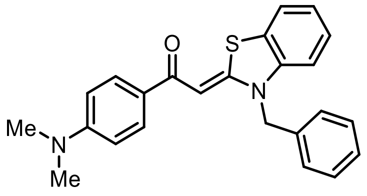

$C_{24}H_{22}N_2OS$

[386.15]

The synthesis was performed according to **GPI** to give 278 mg (0.720 mmol, 72%) of the desired product **3b** as a yellow solid.

Mp: 212 °C.

R_f (*n*-hexane/acetone 4:1): 0.26.

^{1}H NMR (300 MHz, acetone-d_6/CS_2 5:1): δ 3.02, 3.06 (s, 6 H), 5.47 (s, 2 H), 6.62-6.70 (m, 4 H), 7.14-7.38 (m, 7 H), 7.63-7.85 (m, 3 H).

^{13}C NMR (75 MHz, acetone-d_6/CS_2 5:1): δ 40.2 (CH_3), 40.3 (CH_3), 49.7 (CH_2), 88.0 (CH), 110.8 (CH), 110.8 (CH), 111.6 (CH), 111.7 (CH), 111.8 (CH), 122.9 (CH), 123.3 (CH), 127.1 (CH), 127.4 (CH), 128.5 (CH), 129.6 (C_{quat}), 129.7 (C_{quat}), 132.2 (CH), 136.3 (C_{quat}), 141.2 (C_{quat}), 153.1 (C_{quat}), 160.7 (C_{quat}), 183.7 (C_{quat}).

MALDI-TOF (*m/z*): 387.2 ($C_{24}H_{22}N_2OS+H^+$).

IR $\tilde{\nu}$ [cm^{-1}]: 656 (m), 692 (m), 712 (s), 733 (m), 745 (s), 766 (s), 795 (m), 814 (m), 826 (m), 874 (m), 903 (m), 930 (m), 945 (m), 968 (w), 999 (m), 1024 (m), 1047 (m), 1063 (m), 1090 (m), 1125 (m), 1169 (s), 1180 (s), 1234 (m), 1269 (m), 1294 (m), 1341 (m), 1366 (m), 1400 (m), 1425 (m), 1441 (s), 1477 (s), 1530 (m), 1545 (m), 1578 (m), 1612 (m), 1665 (w), 2812 (w), 2859 (w), 2884 (w), 3030 (w), 3059 (w).

UV/Vis (C_3H_6O): λ_{max} (ε) = 404 (60300).

Anal calcd for $C_{24}H_{22}N_2OS$ [386.2]: C 74.58, H 5.74, N 7.25, S 8.29; Found: C 74.43, H 5.89, N 7.25, S 8.42.

(*Z*)-2-(3-(4-Bromobenzyl)benzo[*d*]thiazol-2(3*H*)-ylidene)-1-phenylethan-1-one (3c)

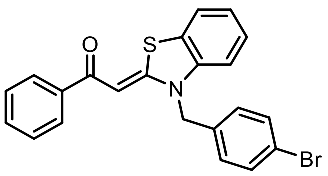

$C_{22}H_{16}BrNOS$

[421.01]

The synthesis was performed according to **GPI** to give 273 mg (0.648 mmol, 65%) of the desired product **3c** as a yellow solid.

Mp: 176 °C (decomposition).

R_f (*n*-hexane/acetone 4:1): 0.18.

^{1}H NMR (300 MHz, acetone-d$_6$): δ 5.57 (s, 2 H), 6.83 (s, 1 H), 7.22-7.27 (m, 3 H), 7.32-7.43 (m, 5 H), 7.51 (d, ^3J = 8.3 Hz, 2 H), 7.73 (d, ^3J = 8.3 Hz, 1 H), 7.93 (d, ^3J = 8.1 Hz, 2 H).

^{13}C NMR (75 MHz, acetone-d$_6$): δ 49.1 (CH_2), 88.2 (CH), 111.2 (CH), 122.2 (C_{quat}), 123.2 (CH), 123.8 (CH), 127.5 (CH), 127.9 (CH), 128.0 (C_{quat}), 128.9 (CH), 129.5 (CH), 131.5 (CH), 132.7 (CH), 135.4 (C_{quat}), 140.4 (C_{quat}), 140.7 (C_{quat}), 162.3 (C_{quat}), 184.3 (C_{quat}).

EI + MS (70 eV, *m/z* (%)): 423 ($[C_{22}H_{16}{}^{81}BrNOS]^+$, 30), 421 ($[C_{22}H_{16}{}^{79}BrNOS]^+$, 30), 406 ($[C_{22}H_{15}{}^{81}BrNS]^+$, 13), 404 ($[C_{22}H_{15}{}^{79}BrNS]^+$, 12), 318 ($[C_{15}H_{11}{}^{81}BrNS]^+$, 25), 316 ($[C_{15}H_{11}{}^{79}BrNS]^+$, 27), 237 (19), 236 ($[C_{15}H_{10}N^{32}S]^+$, 28), 225 (11), 224 (40), 223 ($[C_{14}H_9NS]^+$, 49), 171 ($[C_7H_6{}^{81}Br]^+$, 61), 169 ($[C_7H_6{}^{81}Br]^+$, 62), 105 ($[C_7H_6O]^+$, 100), 90 ($[C_7H_6]^+$, 30), 89 ($[C_7H_5]^+$, 21), 77 ($[C_6H_5]^+$, 29).

IR $\tilde{\nu}$ [cm^{-1}]: 629 (w), 652 (m), 677 (m). 702 (m), 714 (s), 739 (m), 772 (w), 789 (m), 810 (m), 830 (m), 843 (w), 878 (m), 920 (w), 935 (w), 953 (w), 1001 (m), 1045 (m), 1059 (m), 1090 (m), 1111 (w), 1134 (w),1153 (m), 1177 (m), 1198 (m), 1227 (m), 1261 (w), 1275 (w), 1292 (m), 1306 (m), 1341 (m), 1396 (m), 1433 (m), 1449 (m), 1481 (s), 1555 (w), 1566 (m), 1601 (m).

UV/Vis (C_3H_6O): λ_{max} (ε) = 376 (58300).

Anal calcd for $C_{22}H_{16}BrNOS$ [421.1]: C 62.57, H 3.82, N 3.32, S 7.59; Found: C 62.54, H 3.71, N 3.15, S 7.46.

4.2 Synthesis and analytical data of secondary chromophores 4

4.2.1 4-Bromo-*N,N*-bis(4-methoxyphenyl)aniline

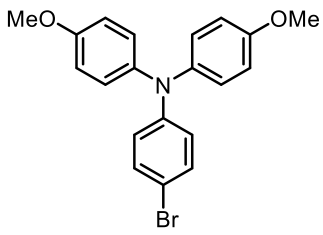

$C_{20}H_{18}BrNO_2$

[383.05]

4-Iodoanisole (2.57 g, 11.0 mmol), 4-bromoaniline (0.956 g, 5.00 mmol), copper(I)-chloride (36.0 mg, 0.200 mmol, 4.00 mol%), 1,10-phenanthroline (36.0 mg, 0.200 mmol, 4.00 mol%) and potassium hydroxide (2.18 g, 39.0 mmol) were placed in a sintered, dry screw-cap *Schlenk*-tube with magnetic stir bar under nitrogen atmosphere and dissolved in 30 mL dry toluene. The solution was degassed with nitrogen for 5 min and then stirred at 120 °C for 20 h. After cooling to rt, 50 mL deionized water and 50 mL dichloromethane were added to the crude product and the mixture was transferred to a separation funnel. The organic layer was separated, and the watery layer was extracted three times with 20 mL dichloromethane. The combined organic layers were dried with anhydrous magnesium sulfate, the drying agent was filtered off und the solvent was removed under reduced pressure. The crude product was adsorbed onto Celite® and purified by flash chromatography on silica gel (*n*-hexane/acetone 100:1). This led to 1.44 g (3.76 mmol, 75 %) of the desired product as an orange solid.

Mp: 99 °C.

R_f (*n*-hexane/acetone 5:1): 0.65.

^{1}H NMR (300 MHz, acetone-d$_6$): δ 3.70 (s, 6 H), 6.70 (d, ^3J = 9.0 Hz, 4 H), 6.73 (d, ^3J = 9.0 Hz, 2 H), 6.94 (d, ^3J = 9.0 Hz, 4 H), 7.14 (d, ^3J = 9.1 Hz, 2 H).

^{13}C NMR (75 MHz, acetone-d$_6$): δ 55.6 (CH_3), 112.5 (C_{quat}), 114.9 (C_{quat}), 122.1 (CH), 126.7 (CH), 131.9 (CH), 138.0 (C_{quat}), 140.7 (C_{quat}), 156.2 (C_{quat}).

MALDI-TOF (*m/z*): 385.13 ($C_{20}H_{18}{}^{81}BrNO_2$), 383.13 ($C_{20}H_{18}{}^{79}BrNO_2$).

4.2.2 4-Methoxy-*N*-(4-methoxyphenyl)-*N*-(4-(4,4,5,5-tetramethyl-1,3,2-dioxaborolan-2-yl)phenyl)aniline (4a)

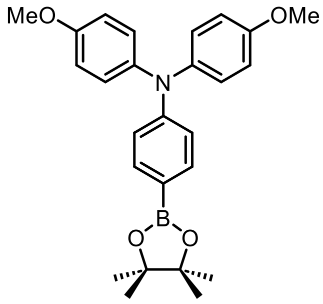

$C_{26}H_{30}BNO_4$

[431.23]

Bromotriphenylamine (1.15 g, 3.00 mmol, 1.00 equiv) was placed in a sintered, dry screw-cap *Schlenk*-tube with magnetic stir bar under nitrogen atmosphere and dissolved in 5 mL dry THF (3 mL/mmol). The reaction mixture was cooled to -78 °C with an acetone/dry ice bath and stirred for 10 min at this temperature. *n*-Butyllithium solution (1.88 mL, 3.00 mmol, 1.00 equiv, 1.6 M in *n*-hexane) was added dropwise. After stirring the reaction mixture at -78 °C for 30 min, tri-*iso*-propylborate (0.710 mL, 3.00 mmol, 1.00 equiv) was added dropwise. After the addition, the mixture was stirred at -78 °C for 10 min and then heated to room temperature. The solution was stirred at room temperature for 30 min before adding pinacol (0.426 mg, 3.60 mmol, 1.20 equivs). The reaction mixture was stirred at room temperature for 69 h. To quench this reaction, concentrated acetic acid (3 mL) were added and the mixture was stirred at room temperature for 1 h. After the addition of 10 mL distilled water and 15 mL dichloromethane, the mixture was transferred to a separation funnel. The organic layer was removed, and the watery phase was extracted three times with 10 mL dichloromethane. The combined organic layers were dried with anhydrous magnesium sulfate, the drying agent was filtered off und the solvent was removed under reduced pressure. The crude product was adsorbed onto Celite® and purified by flash chromatography on silica gel (*n*-hexane/acetone 10:1) to give 1.28 g (2.97 mmol, 99 %) of the desired product **4a** as a colorless solid.

Mp: 128 °C.

R_f (*n*-hexane/ethyl acetate 20:1): 0.17.

^{1}H NMR (300 MHz, acetone-d_6): δ 1.30 (s, 12 H), 3.80 (s, 6 H), 6.84 (d, ^3J = 9.0 Hz, 4 H), 6.88 (d, ^3J = 9.0 Hz, 2 H), 7.07 (d, ^3J = 9.0 Hz, 4 H), 7.61 (d, ^3J = 9.1 Hz, 2 H).

^{13}C NMR (75 MHz, acetone-d$_6$): δ 25.0 (CH$_3$), 55.6 (CH$_3$), 83.5 (C$_{quat}$), 114.8 (CH), 118.8 (CH), 127.3 (CH), 135.9 (CH), 140.5 (C$_{quat}$), 151.5 (C$_{quat}$), 156.3 (C$_{quat}$). (The quaternary carbon nucleus covalently bound to the boron core could not be observed in the ^{13}C NMR spectrum.)

EI + MS (70 eV, *m/z* (%)): 432 (27), 431 ([$C_{26}H_{30}BNO_4$]$^+$, 100), 430 (24), 417 (13), 416 ([$C_{25}H_{27}BNO_4$]$^+$, 52), 415 (13), 316 ([$C_{20}H_{18}BNO_2$]$^+$, 24), 305 ([$C_{20}H_{18}NO_2$]$^+$, 18), 290 ([$C_{17}H_{12}N$]$^+$, 14), 57 ([C_3H_5O]$^+$, 19), 56 ([C_3H_4O]$^+$, 28).

4.2.3 5-(4,4,5,5-Tetramethyl-1,3,2-dioxaborolan-2-yl)-2,2'-bipyridine (4b)

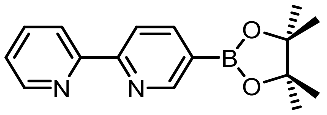

$C_{16}H_{19}BN_2O_2$

[282.15]

5-Bromo-2,2‘-bipyridine (0.470 g, 2.00 mmol, 1.00 equiv) was placed in a sintered, dry screw-cap *Schlenk*-tube with magnetic stir bar under nitrogen atmosphere and dissolved in 10 mL DMSO. Potassium acetate (1.02 g, 10.0 mmol, 5.00 equivs), bisp(pinacolato)diboron (0.533 g, 2.10 mmol, 1.05 equivs) and bis(triphenylphosphane)palladium(II)-dichloride (0.140 g, 0.01 mmol) were added and the brown reaction mixture was stirred at 80 °C for 19 h. The catalysator was filtered off and 20 mL chloroform was added to the crude mixture. The mixture was transferred to a separation funnel and the organic layer was extracted five times with 50 mL deionized water. The combined organic layers were dried with anhydrous magnesium sulfate, the drying agent was filtered off und the solvent was removed under reduced pressure. The crude product was adsorbed onto Celite® and purified by flash chromatography on silica gel (*n*-hexane/acetone 8:1 to 4:1) to give 0.118 g (0.418 mmol, 21%) of the desired product as a colorless solid.

Mp: 87 °C.

R$_f$ (*n*-hexane/acetone 4:1): 0.22.

^{1}H NMR (300 MHz, CDCl$_3$): δ 1.30 (s, 12 H), 7.28 – 7.30 (m, 1 H), 7.76 – 7.82 (m, 1 H), 8.01 – 8.05 (m, 1 H), 8.38 – 8.50 (m, 2 H), 8.64 – 8.66 (m, 1 H), 8.92 – 8.95 (m, 1 H).

^{13}C NMR (75 MHz, CDCl$_3$): δ 25.0 (CH$_3$), 84.4 (C$_{quat}$), 121.4 (CH), 124.1 (CH), 133.3 (CH), 135.3 (CH), 137.3 (CH), 147.6 (CH), 149.4 (CH), 155.6 (C$_{quat}$), 155.8 (C$_{quat}$). (The quaternary carbon nucleus covalently bound to the boron core could not be observed in the ^{13}C NMR spectrum.)

EI + MS (70 eV, *m/z* (%)): 282 ($[C_{16}H_{19}BN_2O_2]^+$, 42), 281 (12), 267 ($[C_{15}H_{16}BN_2O_2]^+$, 12), 266 (29), 239 (10), 225 ($[C_{12}H_{10}BN_2O_2]^+$, 17), 196 ($[C_{10}H_6BN_2O_2]^+$, 17), 185 (31), 184 (15), 183 (52), 182 ($[C_{10}H_7BN_2O]^+$, 43), 180 (20), 156 (12), 155 ($[C_{10}H_7N_2]^+$, 18), 154 (10), 129 ($[C_6H_{14}B_2]^+$, 68), 128 (27), 103 (25), 102 (10), 101 ($[C_7H_3N]^+$, 12), 85 (33), 84 ($[C_6H_{12}]^+$, 64), 83 (26), 59 ($[C_3H_7O]^+$, 100), 58 (14), 57 (29), 55 (13).

4.3 Synthesis and analytical data of bi- and multichromophores 5

4.3.1 (*Z*)-2-(3-((4'-(Bis(4-methoxyphenyl)amino)-[1,1'-biphenyl]-4-yl)methyl)-benzo[*d*]thiazol-2(3*H*)-ylidene)-1-phenylethan-1-one (5a)

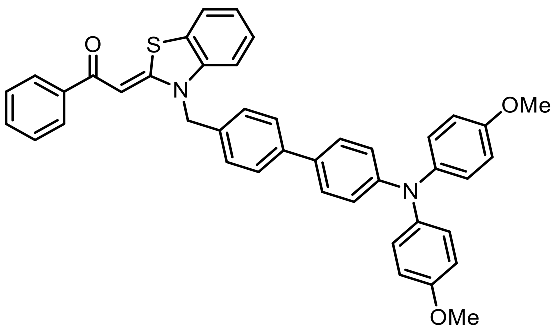

$C_{42}H_{34}N_2O_3S$

[646.23]

Benzoylchloride **1b** (0.07 g, 0.50 mmol, 1.00 equiv) and 4-benzylbenzothiazoliumbromide **2b** (0.219 g, 0.550 mmol, 1.10 equivs) were placed in a sintered, dry screw-cap *Schlenk*-tube with magnetic stir bar under nitrogen atmosphere and dissolved in 3 mL dry 1,4-dioxane and 1 mL ethanol. Triethylamine as a base (0.150 mL, 1.10 mmol, 2.20 equivs) was added to the reaction mixture and the solution was stirred for 1 h at room temperature. Thereafter, the reaction mixture was stirred at 120 °C for 20 h. After cooling to room temperature tetrakis(triphenylphosphane)palladium(0) (15.0 mg, 2.00 mol%, 0.0125 mmol), triphenylamine boronic acid ester **4a** (0.431 g, 1.00 mmol, 2.00 equivs) and cesium carbonate (0.480 g, 1.50 mmol, 3.00 equivs) were added to the reaction mixture under nitrogen atmosphere and the mixture was stirred at 120 °C (oil bath) for 4 h. The crude product was adsorbed onto Celite® and purified by flash chromatography on silica gel (*n*-hexane/acetone 6:1 to 4:1 to 2:1). The product was suspended in *n*-hexane, the sediment was filtrated and dried under vacuo. It was possible to isolate 0.214 g (0.331 mmol, 66 %) of the desired product **5a** as a yellow solid.

Mp: 148 °C.

R_f (*n*-hexane/acetone 4:1): 0.14.

^{1}H NMR (300 MHz, acetone-d$_6$): δ 3.75 (s, 6 H), 5.58 (s, 2 H), 6.84-6.89 (m, 6 H), 6.96 (s, 1 H), 6.99-7.03 (m, 4 H), 7.18-7.24 (m, 1 H), 7.30-7.42 (m, 10 H), 7.54 (d, ^3J = 8.3 Hz, 1 H), 7.75 (d, ^3J = 8.3 Hz, 1 H), 7.99 (dd, ^3J = 7.4 Hz, ^4J = 1.8 Hz, 2 H).

^{13}C NMR (75 MHz, acetone-d$_6$): δ 49.2 (CH_2), 55.68 (CH_3), 55.70 (CH_3), 88.2 (CH), 111.5 (CH), 115.6 (CH), 120.8 (CH), 123.2 (CH), 123.8 (CH), 127.35 (CH), 127.43 (CH), 127.5 (CH), 127.7 (CH), 127.8 (C_{quat}), 127.9 (CH), 128.0 (CH), 128.1 (CH), 129.1 (C_{quat}), 131.6 (C_{quat}), 132.6 (CH), 134.3 (C_{quat}), 140.6 (C_{quat}), 140.9 (C_{quat}), 141.4 (C_{quat}), 149.3 (C_{quat}), 157.2 (C_{quat}), 162.6 (C_{quat}), 184.5 (C_{quat}).

MALDI-TOF (*m/z*): 646.3 ($C_{42}H_{34}N_2O_3S^+$).

IR $\tilde{\nu}$ [cm^{-1}]: 669 (m), 691 (m), 702 (m), 720 (m), 743 (m), 816 (m), 828 (m), 880 (m), 1001 (m), 1034 (m), 1061 (m), 1074 (m), 1103 (m), 1177 (m), 1194 (m), 1236 (s), 1285 (m), 1319 (m), 1400 (m), 1437 (s), 1452 (s), 1570 (m), 1603 (m), 2832 (m), 2901 (m), 2961 (m), 2990 (m).

UV/Vis (C_3H_6O): λ_{max} (ε) = 340 (sh), 373 (39400).

Anal calcd for $C_{42}H_{34}N_2O_3S$ [646.2]: C 77.99, H 5.30, N 4.33, S 4.96; Found: C 77.80, H 5.49, N 4.14, S 4.66.

4.3.2 (*Z*)-2-(3-(4-([2,2'-Bipyridine]-5-yl)benzyl)benzo[*d*]thiazol-2(3*H*)-ylidene)-1-(4-(dimethylamino)phenyl)ethan-1-one (5b)

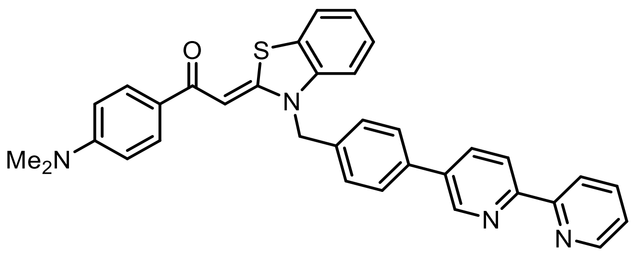

$C_{34}H_{28}N_4OS$

[540.20]

Dimethylamino aroyl-*S,N*-ketene acetal (**3a**), (0.085 g., 0.300 mmol, 1.00 equiv), bipyridine boronic acid ester (**4b**) (0.139 g,.0.300 mmol, 1.00 equiv), cesium carbonate (0.293 g, 0.900 mmol, 3.00 equivs) and tetrakis(triphenylphosphane)palladium(0) (0.015 g, 0.006 mmol, 2.00 mol%) were placed in a sintered, dry screw-cap *Schlenk*-tube with magnetic stir bar under nitrogen atmosphere and dissolved in 2 mL dry 1,4-dioxane and 1 mL ethanol. The mixture was stirred at 120 °C (oil bath) for 4 h. The crude product was adsorbed onto Celite® and purified by flash chromatography on silica gel (*n*-hexane/acetone 3:1 to 1:1 to pure acetone). The product was suspended in *n*-hexane, the sediment was filtrated and dried under vacuo. It was possible to isolate 0.157 g (0.291 mmol, 97%) of the desired product **5b** as a yellow solid.

Mp: 190 °C.

R_f (*n*-hexane/acetone 3:1): 0.19.

^{1}H NMR (300 MHz, acetone-d$_6$/CS$_2$ 5:1): δ 3.03, 3.03 (s, 6 H), 5.47 (s, 2 H), 6.63 – 6.76 (m, 3 H), 7.16 – 7.30 (m, 1 H), 7.38 – 7.42 (m, 2 H), 7.52 (d, ^3J = 8.6 Hz, 1 H), 7.64 – 7.67 (m, 1 H), 7.77 – 7.92 (m, 2 H), 8.07 (dd, ^3J = 8.6 Hz, ^4J = 2.4 Hz, 2 H), 8.41 – 8.44 (m, 4 H), 8.65 (dq, ^3J = 4.7 Hz, ^4J = 1.0 Hz, 2 H), 8.07 (dd, ^4J = 2.3 Hz, ^4J = 0.6 Hz, 2 H).

^{13}C NMR (75 MHz, acetone-d$_6$/CS$_2$ 5:1): δ 40.2 (CH_3), 40.3 (CH_3), 48.9 (CH_2), 87.9 (CH), 110.7 (CH), 111.7 (CH), 121.4 (CH), 121.8 (CH), 122.1 (C_{quat}), 122.9 (CH), 123.1 (CH), 123.4 (CH), 125.0 (CH), 127.1 (CH), 129.2 (C_{quat}), 128.3 (C_{quat}), 129.5 (CH), 129.6 (CH), 132.1 (C_{quat}), 132.7 (CH), 132.9 (CH), 135.6 (C_{quat}), 137.7 (CH), 140.2 (CH), 140.9 (C_{quat}), 150.0 (CH), 150.7 (CH), 153.0 (C_{quat}), 155.4 (C_{quat}), 155.6 (C_{quat}), 160.5 (C_{quat}), 183.7 (C_{quat}).

ESI-MS (m/z): 541 ($C_{34}H_{28}N_4OS + H^+$).

IR $\tilde{\nu}$ [cm^{-1}]: 652 (w), 694 (m), 719 (m), 746 (s), 768 (m), 793 (m), 810 (w), 826 (m), 856 (w), 874 (m), 910 (w), 930 (w), 947 (m), 972 (w), 989 (m), 1022 (m), 1043 (m), 1063 (m), 1090 (m), 1119 (m), 1169 (s), 1180 (m), 1236 (m), 1265 (w), 1294 (m), 1310 (w), 1342 (m), 1366 (m), 1408 (w), 1423 (m), 1439 (m), 1466 (m), 1477 (s), 1526 (m), 1543 (m), 1574 (m), 1811 (w), 2625 (w), 2814 (w), 2853 (w), 2903 (w), 2922 (w), 3455 (w).

UV/Vis (C_3H_6O): λ_{max} (ε) = 404 (51600).

Anal calcd for $C_{34}H_{28}N_4OS$ [540.2]: C 75.53, H 5.22, N 10.36, S 5.93; Found.: C 75.60, H 5.09, N 9.97, S 5.87.

4.3.3 (2*Z*,2'*Z*)-2,2'-((((1,10-Phenanthrolin-3,8-diyl)bis(4,1-phenylene))bis(methylene))bis(benzo[*d*]thiazol-3(3*H*)-yl-2(3*H*)-ylidene))bis(1-(4-(dimethylamino)phenyl)ethan-1-one) (5c)[9]

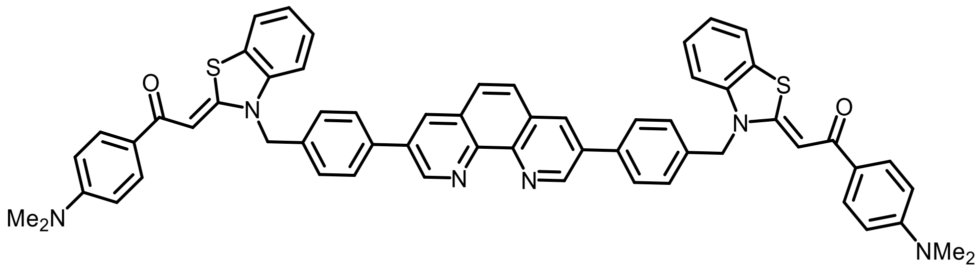

$C_{60}H_{48}N_6O_2S_2$

[948.33]

Dimethylamino aroyl-*S,N*-ketene acetal (0.278 g, 0.600 mmol, 1.20 equivs) and tetrakis(triphenylphosphane)palladium(0) (0.058 g, 0.050 mmol, 20.0 mol%) were placed in a sintered, dry screw-cap *Schlenk*-tube with magnetic stir bar under nitrogen atmosphere and dissolved in 3 mL dry 1,4-dioxane. Pinacolborane (0.145 mL, 1.00 mmol, 4.00 equivs) and triethylamine (0.680 mL, 5.00 mmol, 20.0 equivs) were added to the reaction mixture and the solution was stirred at 120 °C (oil bath) for 20 h. After cooling to room temperature, 1,10-dibromophenanthroline (0.084 g, 0.250 mmol, 1.00 mmol), cesium carbonate (0.408 g, 1.25 mmol, 2.50 equivs) and 1 mL methanol were added to the reaction mixture under nitrogen atmosphere and the mixture was stirred at 120 °C (oil bath) for 22 h. The crude product was adsorbed onto Celite® and purified by flash chromatography on silica gel (*n*-hexane/acetone 2:1 to 1:2 to 1:4 to acetone to acetone + 2 % methanol). The product was suspended in *n*-hexane, the sediment was filtrated and dried under vacuo to give compound **5c** (0.116 mg, 0.122 mmol, 49%) as an orange solid.

Mp: 166 °C.

R_f (*n*-hexane/acetone 1:1): 0.19.

^{1}H NMR (500 MHz, DMSO-d$_6$): δ 2.97, 2.98 (s, 12 H), 5.56 (s, 4 H), 6.71 (d, ^3J = 8.4 Hz, 4 H), 6.77–6.82 (m, 4 H), 7.17-7.25 (m, 5 H), 7.33-7.40 (m, 3 H), 7.54-7.56 (m, 3 H), 7.75-7.78 (m, 2 H), 7.82-7.85 (m, 5 H), 8.01 (s, 2 H), 8.84 (d, ^3J = 2.5 Hz, 2 H), 9.15 (s, 2 H).

^{13}C NMR (125 MHz, DMSO-d$_6$): δ 39.9 (CH$_3$), 40.0 (CH$_3$), 47.6 (CH$_2$), 87.2 (CH), 110.5 (CH), 110.9 (CH), 111.2 (CH), 120.6 (C$_{quat}$), 122.4 (CH), 122.6 (CH), 126.4 (CH), 126.6 (C$_{quat}$), 127.3 (CH), 128.7 (CH), 129.0 (CH), 129.8 (C$_{quart}$), 130.9 (CH), 131.7 (CH), 132.1 (CH), 135.3 (C$_{quat}$), 137.9 (CH), 139.8 (C$_{quat}$), 143.6 (C$_{quat}$), 150.8 (C$_{quat}$), 152.2 (C$_{quat}$), 159.5 (C$_{quat}$), 167.5 (C$_{quat}$), 182.7 (C$_{quat}$).

MALDI-TOF (m/z): 949 ($C_{60}H_{48}N_6O_2S + H^+$).

IR $\tilde{\nu}$ [cm^{-1}]: 633 (m), 696 (m), 729 (m), 770 (m), 789 (m), 827 (m), 872 (w), 881 (w), 914 (w), 945 (m), 1001 (w), 1038 (m), 1059 (m), 1119 (m), 1165 (s), 1231 (m), 1287 (m), 1317 (m), 1366 (m), 1418 (m),1470 (m), 1504 (m), 1524 (m), 1557 (m), 1595 (s), 1634 (w), 1638 (w), 1667 (w), 1703 (w), 2919 (w), 2929 (w), 2976 (w), 3055 (w), 3452 (w).

UV/Vis (C_3H_6O): λ_{max} (ε) = 373 (25400).

Anal calcd for $C_{60}H_{48}N_6O_2S_2$ [948.3]: C 75.92, H 5.10, N 8.85, S 6.76; Found: C 75.70, H 5.17, N 8.65, S 6.64.

5 NMR spectra

^{1}H NMR-spectrum of (*Z*)-2-(3-(4-bromobenzyl)benzo[*d*]thiazol-2(3*H*)-ylidene)-1-(4-dimethylaminophenyl)ethan-1-one (3a) (acetone-d_6/CS_2 5:1, 300 MHz, 293 K)[4]

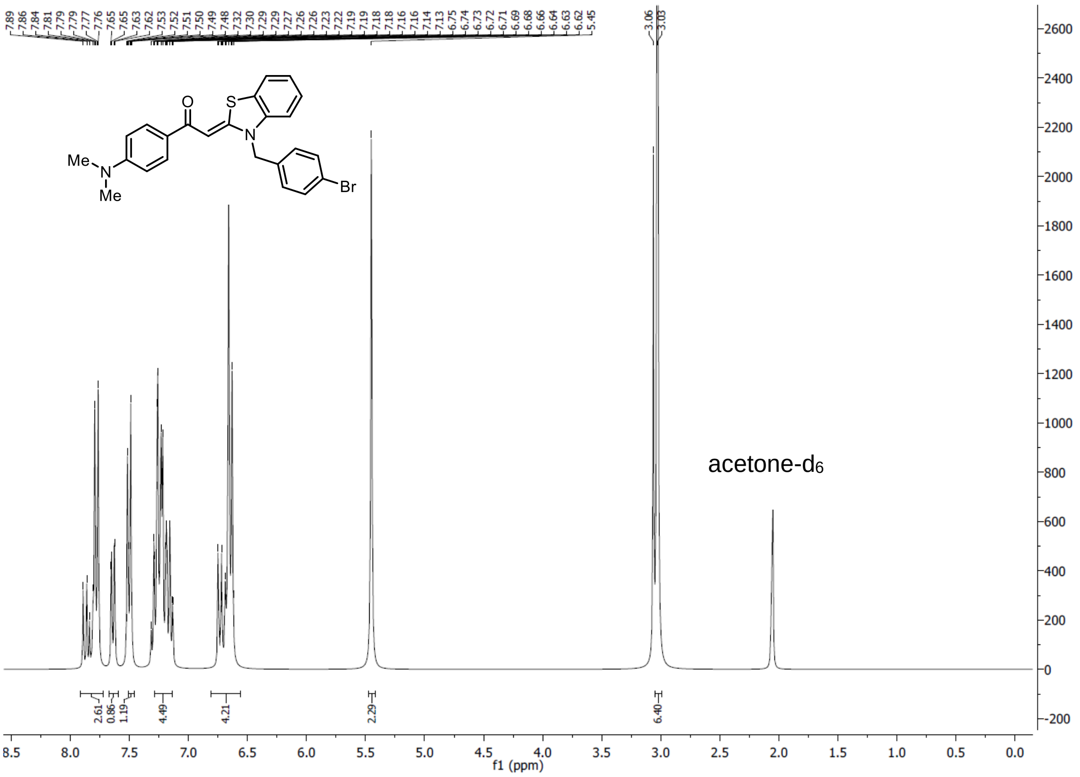

^{13}C NMR-spectrum of (*Z*)-2-(3-(4-bromobenzyl)benzo[*d*]thiazol-2(3*H*)-ylidene)-1-(4-dimethylaminophenyl)ethan-1-one (3a) (acetone-d_6/CS_2 5:1, 75 MHz, 293 K)[4]

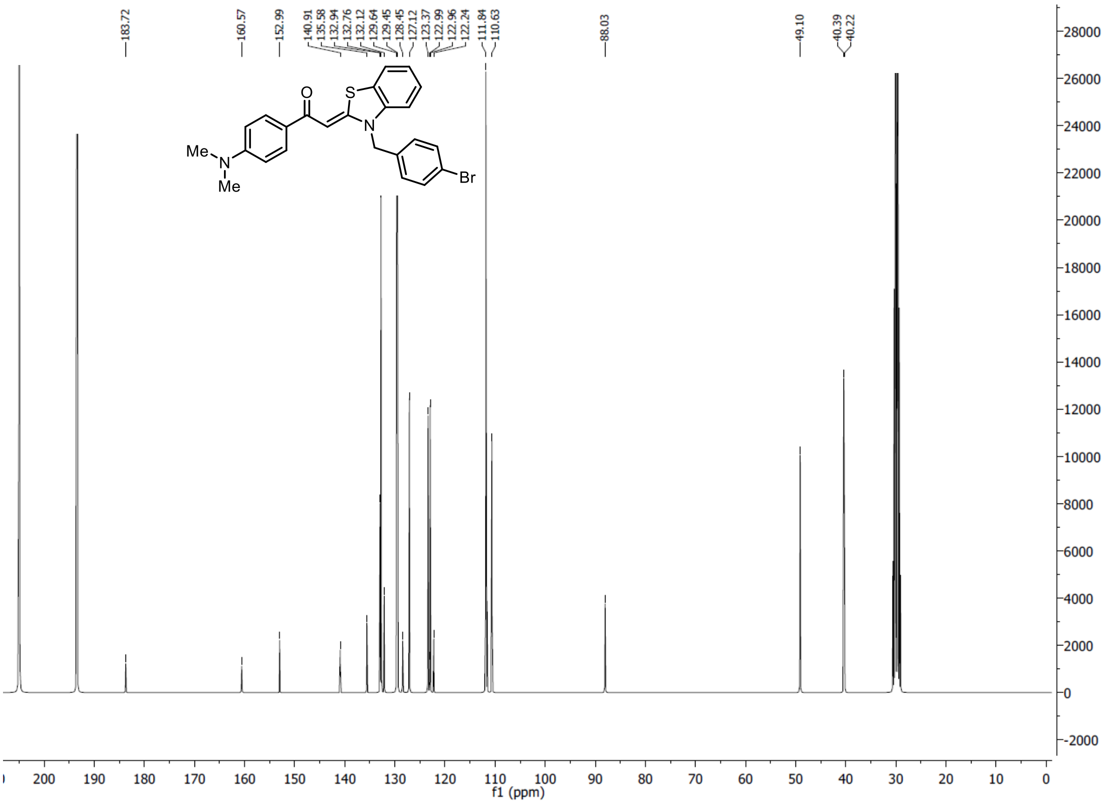

^{1}H NMR-spectrum of (*Z*)-2-(3-benzyl)benzo[*d*]thiazol-2(3*H*)-ylidene)-1-(4-dimethylaminophenyl)ethan-1-one (3b) (acetone-d_6/CS_2 5:1, 300 MHz, 293 K)[4]

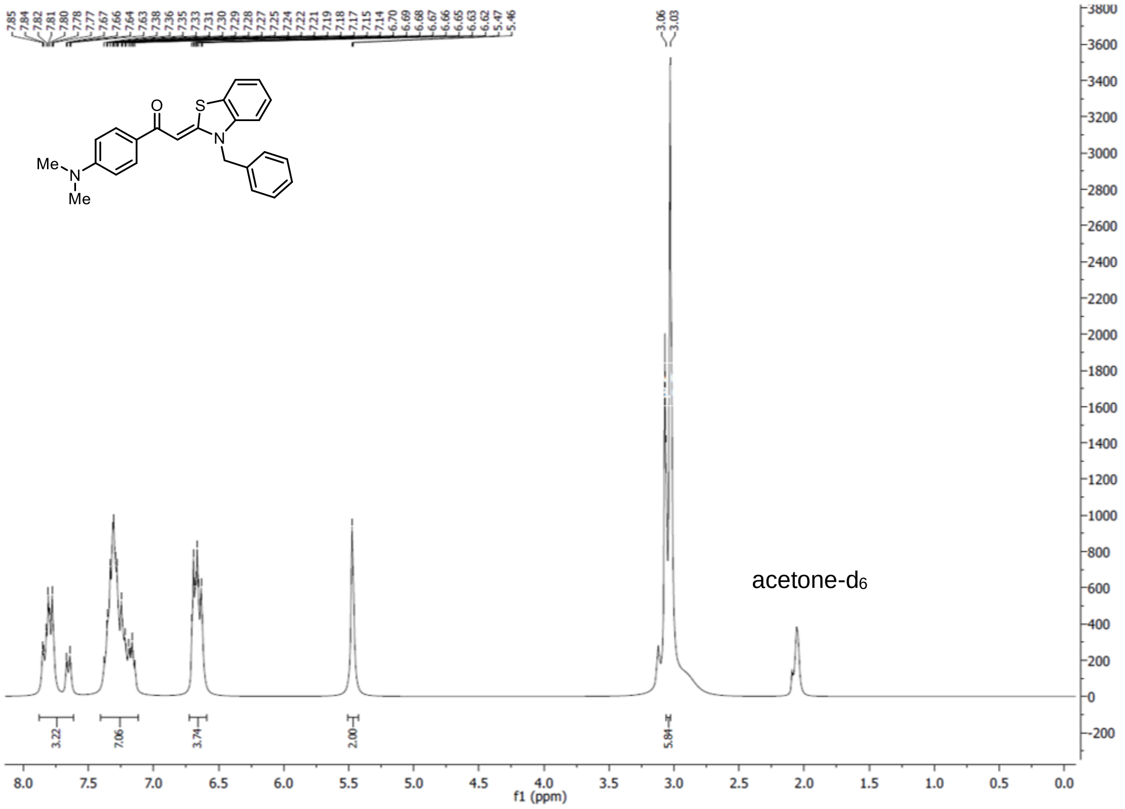

^{13}C NMR-spectrum von (*Z*)-2-(3-benzyl)benzo[*d*]thiazol-2(3*H*)-ylidene)-1-(4-dimethylaminophenyl)ethan-1-one (3b) (acetone-d_6/CS_2 5:1, 75 MHz, 293 K)[4]

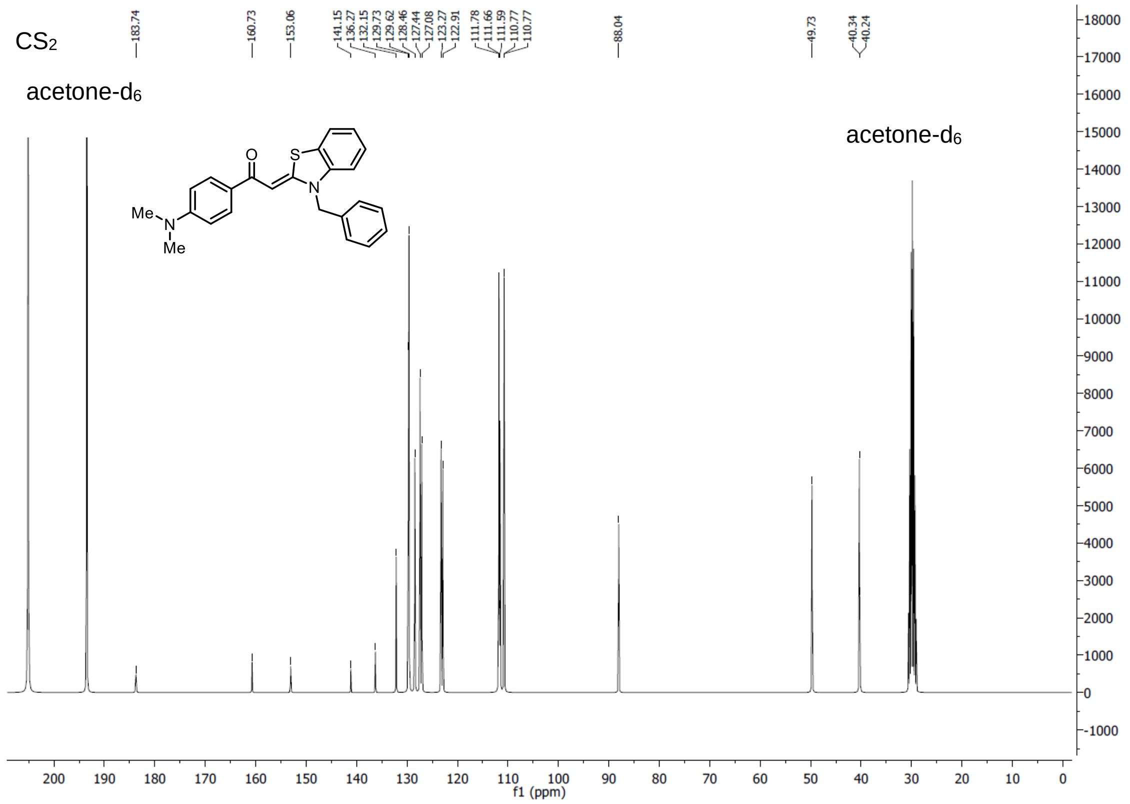

^{1}H NMR-spectrum of (*Z*)-2-(3-(4-bromobenzyl)benzo[*d*]thiazol-2(3*H*)-ylidene)-1-phenylethan-1-one (3c) (acetone-d_6/CS_2 5:1, 300 MHz, 293 K)[4]

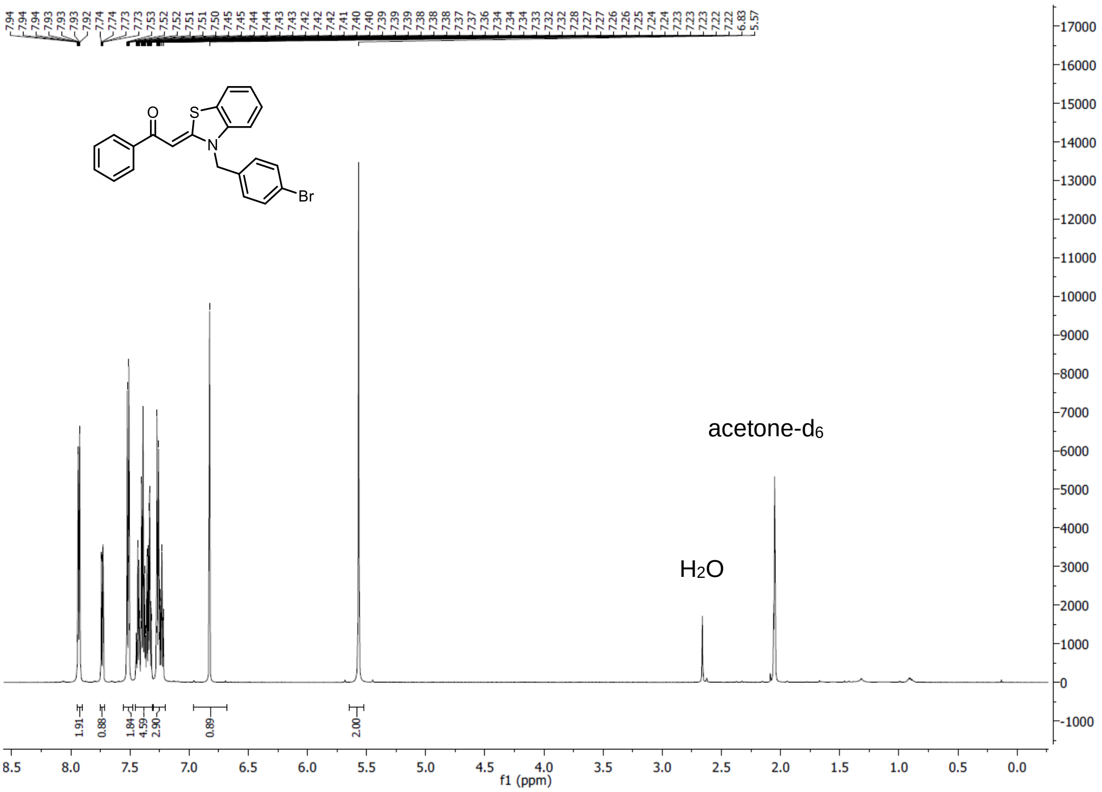

^{13}C NMR-spectrum of (*Z*)-2-(3-(4-bromobenzyl)benzo[*d*]thiazol-2(3*H*)-ylidene)-1-phenylethan-1-one (3c) (acetone-d_6/CS_2 5:1, 75 MHz, 293 K)[4]

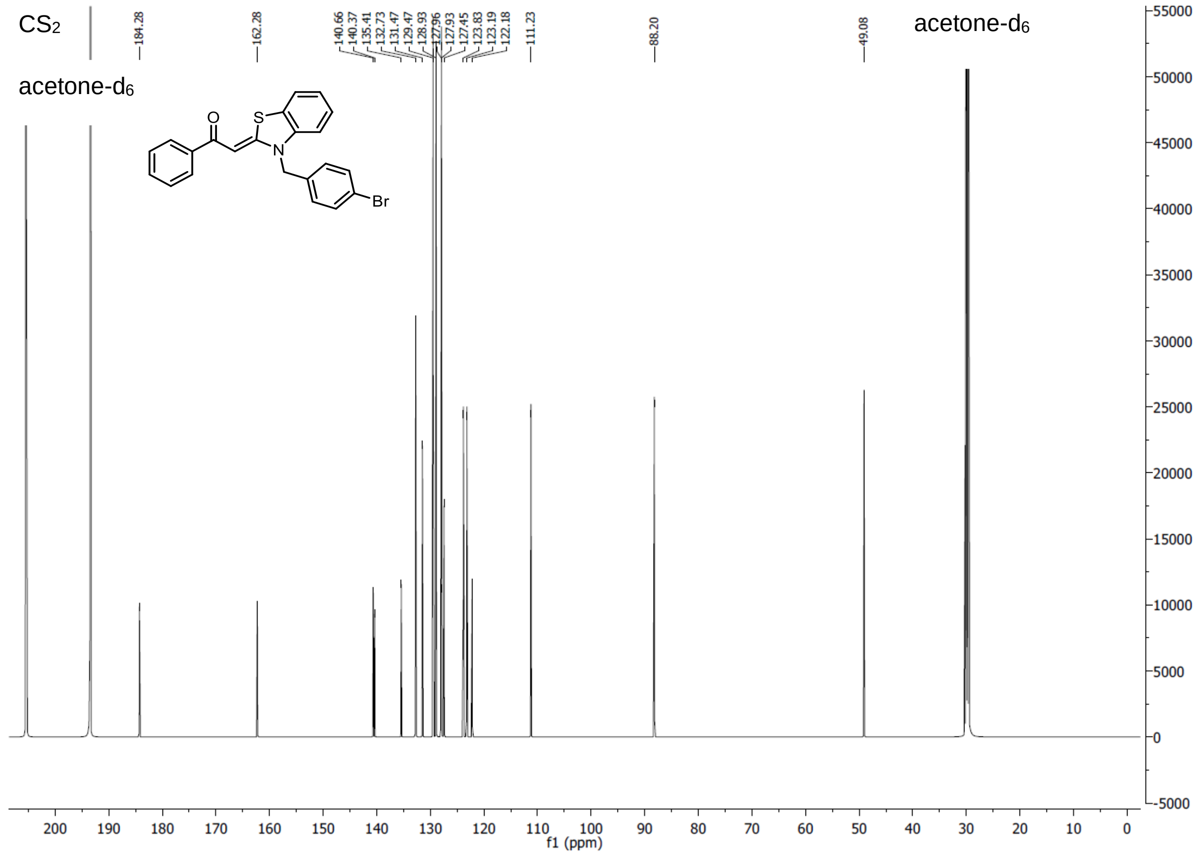

^{1}H NMR-spectrum of (*Z*)-2-(3-((4'-(bis(4-methoxyphenyl)amino)-[1,1'-biphenyl]-4-yl)methyl)benzo[*d*]thiazol-2(3*H*)-ylidene)-1-phenylethan-1-one (5a) (acetone-d$_6$, 300 MHz, 293 K)

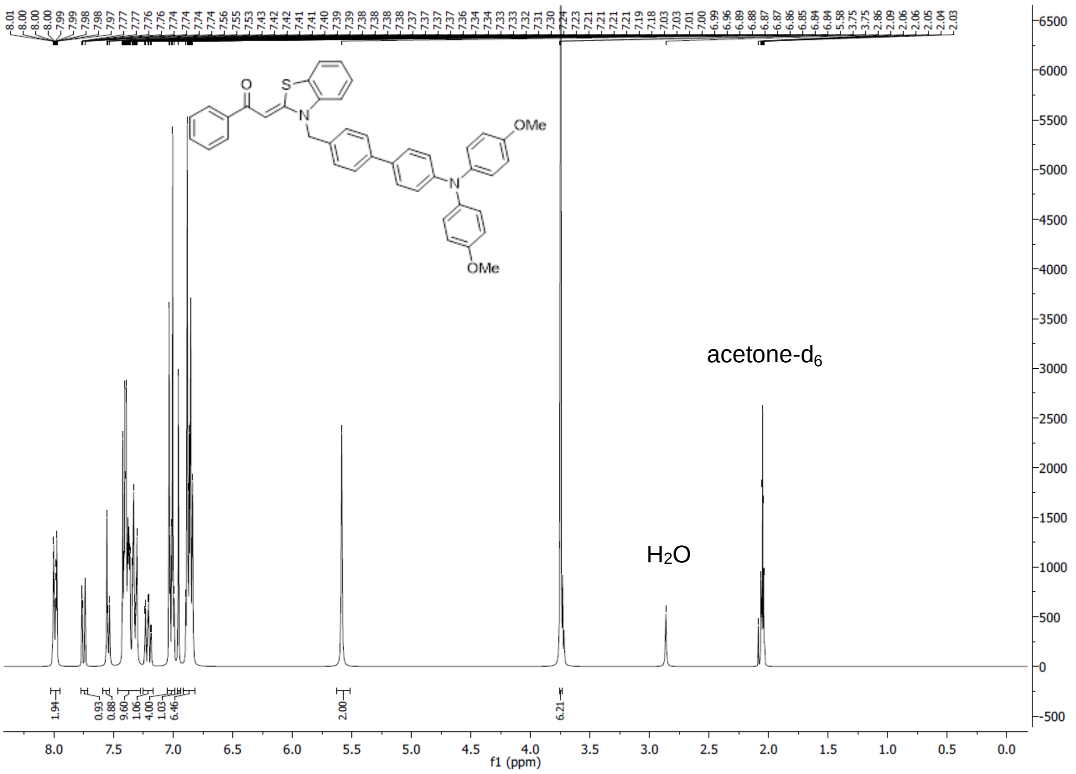

^{13}C NMR-spectrum of (*Z*)-2-(3-((4'-(bis(4-methoxyphenyl)amino)-[1,1'-biphenyl]-4-yl)methyl)benzo[*d*]thiazol-2(3*H*)-ylidene)-1-phenylethan-1-one (5a) (acetone-d$_6$, 75 MHz, 293 K)

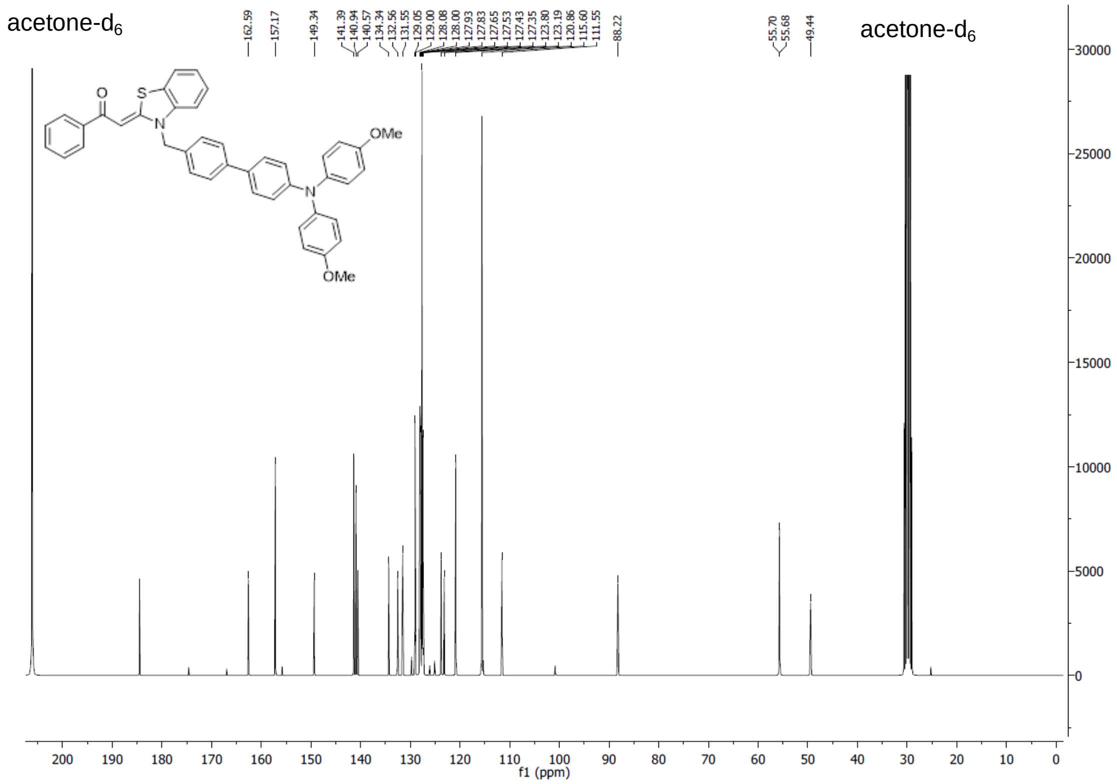

¹H NMR-Spektrum (*Z*)-2-(3-(4-([2,2'-bipyridine]-5-yl)benzyl)benzo[*d*]thiazol-2(3*H*)-ylidene)-1-(4-(dimethylamino)phenyl)ethan-1-one (5b) (300 MHz, acetone-d_6/CS_2 5:1, 293 K)

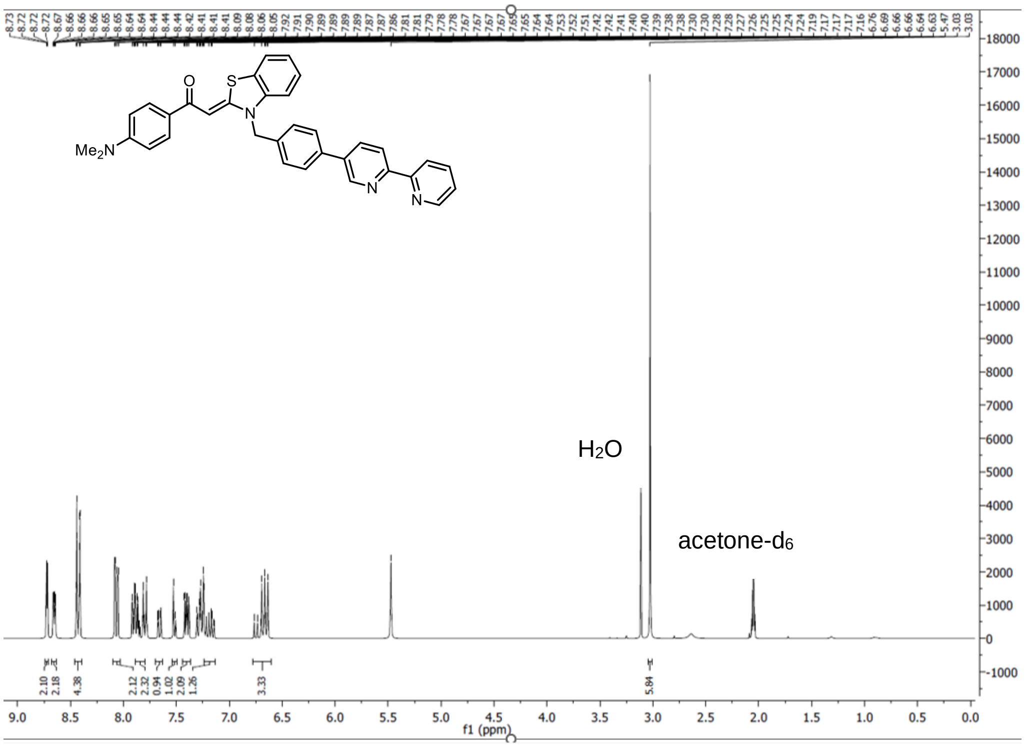

¹³C NMR-Spektrum (*Z*)-2-(3-(4-([2,2'-bipyridine]-5-yl)benzyl)benzo[*d*]thiazol-2(3*H*)-ylidene)-1-(4-(dimethylamino)phenyl)ethan-1-one (5b) (75 MHz, acetone-d_6/CS_2 5:1, 293 K)

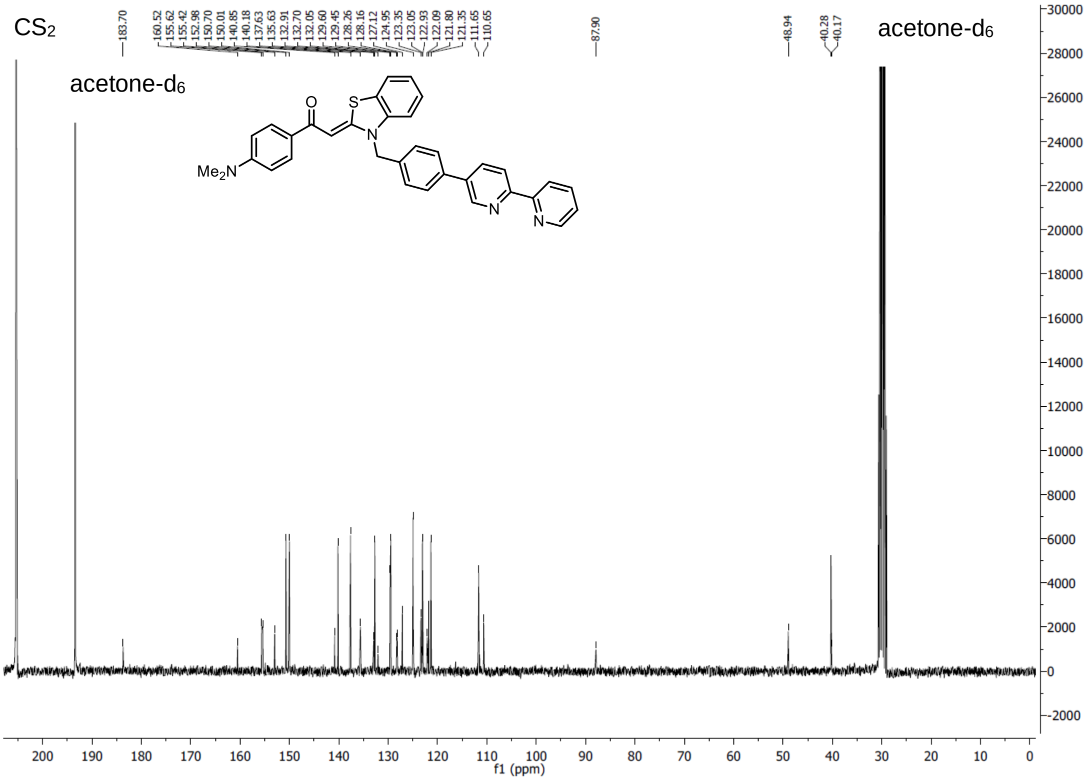

^{1}H NMR spectrum (2*Z*,2'*Z*)-2,2'-((((1,10-phenanthrolin-3,8-diyl)bis(4,1-phenylene))bis(methylene))bis(benzo[*d*]thiazol-3(3*H*)-yl-2(3*H*)-ylidene))bis(1-(4-(dimethylamino)phenyl)ethan-1-one) (5c) (500 MHz, DMSO-d$_6$, 293 K)[9]

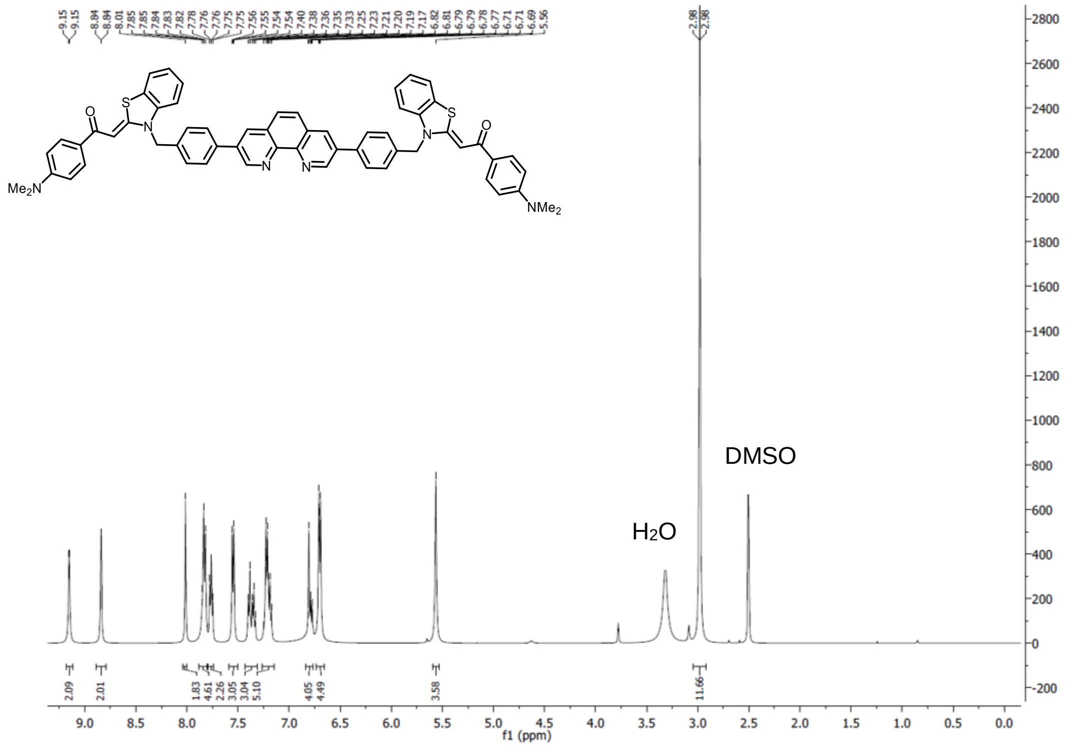

^{13}C NMR spectrum (2*Z*,2'*Z*)-2,2'-((((1,10-phenanthrolin-3,8-diyl)bis(4,1-phenylene))bis(methylene))bis(benzo[*d*]thiazol-3(3*H*)-yl-2(3*H*)-ylidene))bis(1-(4-(dimethylamino)phenyl)ethan-1-one) (5c) (125 MHz, DMSO-d$_6$, 293 K)[9]

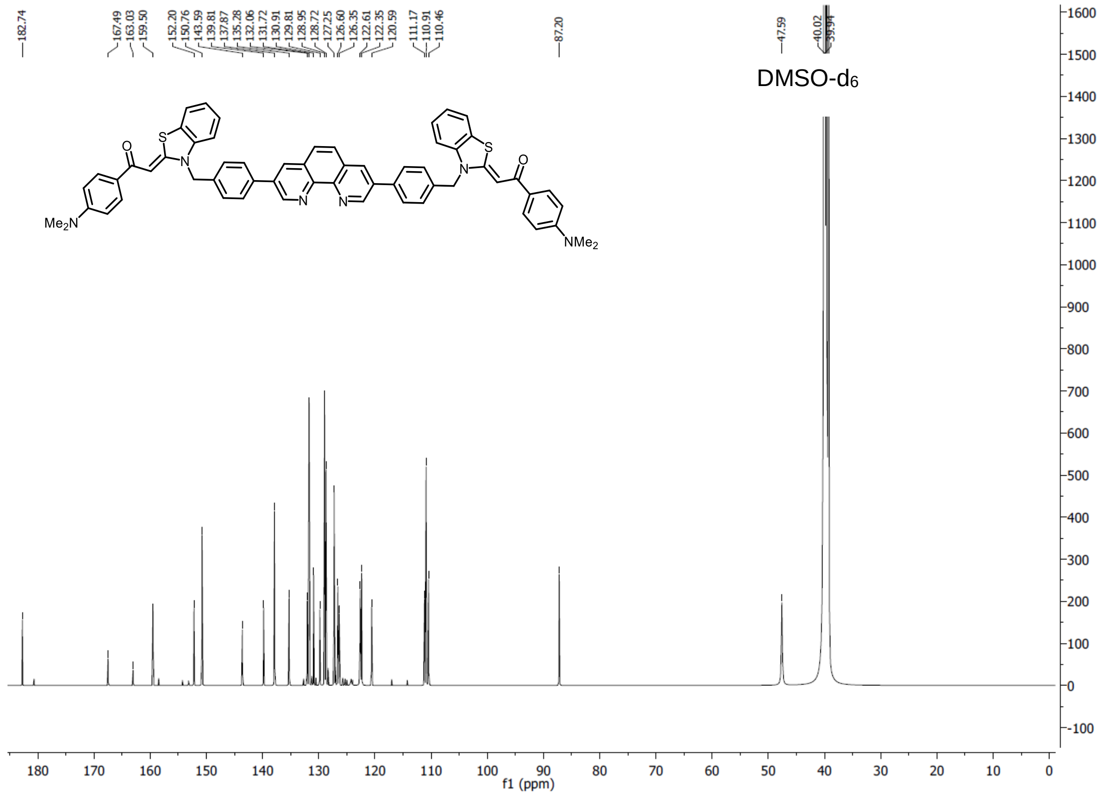

6 Overview of photophysical properties of aroyl-*S,N*-ketene acetal metal sensors 3 and 5

All solution spectra were recorded in ethanol or in ethanol/water mixtures at T = 298 K, the excitation wavelengths λ_{exc} for the AIE-titration studies and the emission spectra in solution were determined from the absorption maxima λ_{max} of this compound, the excitation wavelength for the solid-state emission spectra was determined from solid state excitation spectra. The dye concentration of the solution for absorption measurements was c = 10^{-5} M and the dye concentration of the ethanol/water mixtures for AIE measurements was c = 10^{-7} M.

Table S3: Photophysical properties of investigated aroyl-*S,N*-ketene acetal metal sensors.

Entry	Example	$\lambda_{max(abs.)}$ solution [nm] (ε [L·mol^{-1}·cm^{-1}])[a]	$\lambda_{max(em.)}$ solution [nm][b]	*Stokes* shift[c[$\tilde{\nu}$ [cm^{-1}]	$\lambda_{max(em.)}$ solid-state [nm][d]	solid-state[e]
1	**3a**	404 (57700)	455	24800	604	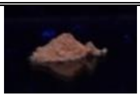
2	**3b**	404 (60300)	455	24800	534	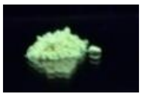
3	**5a**	330 (sh), 373 (43600)	447	4430	500	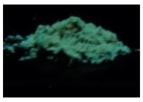
4	**5b**	404 (51600)	455	2770	604	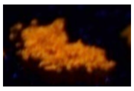
5	**5c**	274 (26300) 373 (25400)	455	4800	571	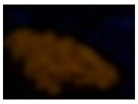

[a]: measured in ethanol, T = 298 K, c(**5**) = 10^{-5} M, [b]: measured in ethanol, T = 298 K, c(**5**) = 10^{-7} M, λ_{exc} = $\lambda_{abs,max}$ if not otherwise specified, [c]: $\tilde{\nu} = \tilde{\nu}_{max(Abs.)} - \tilde{\nu}_{max(Em.)}$, [d]: T = 298 K, λ_{exc} = $\lambda_{abs,max}$, [e]: pictures taken under UV-light (λ_{exc} = 365 nm).

7 Absorption and emission spectra

All solution spectra were recorded in ethanol or in ethanol/water mixtures at T = 298 K, the excitation wavelengths λ_{exc} for the AIE-titration studies and the emission spectra in solution were determined from the absorption maxima λ_{max} of this compound, the excitation wavelength for the solid-state emission spectra was determined from solid state excitation spectra. The dye concentration of the solution for absorption measurements was $c = 10^{-5}$ M and the dye concentration of the ethanol/water mixtures for AIE measurements was $c = 10^{-7}$ M.

7.1 Absorption and emission spectra of aroyl-*S,N*-ketene acetal metal sensors

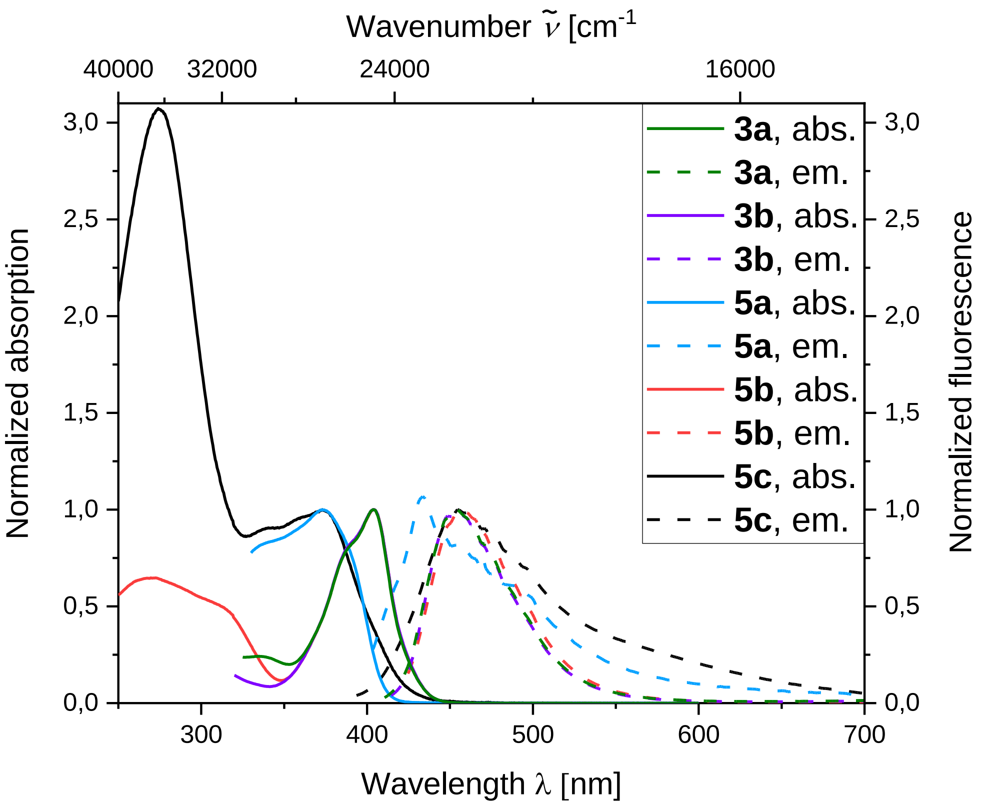

Figure S1: Normalized absorption and emission bands of aroyl-*S*,*N*-ketene acetal metal sensors **3** and **5** in ethanol.

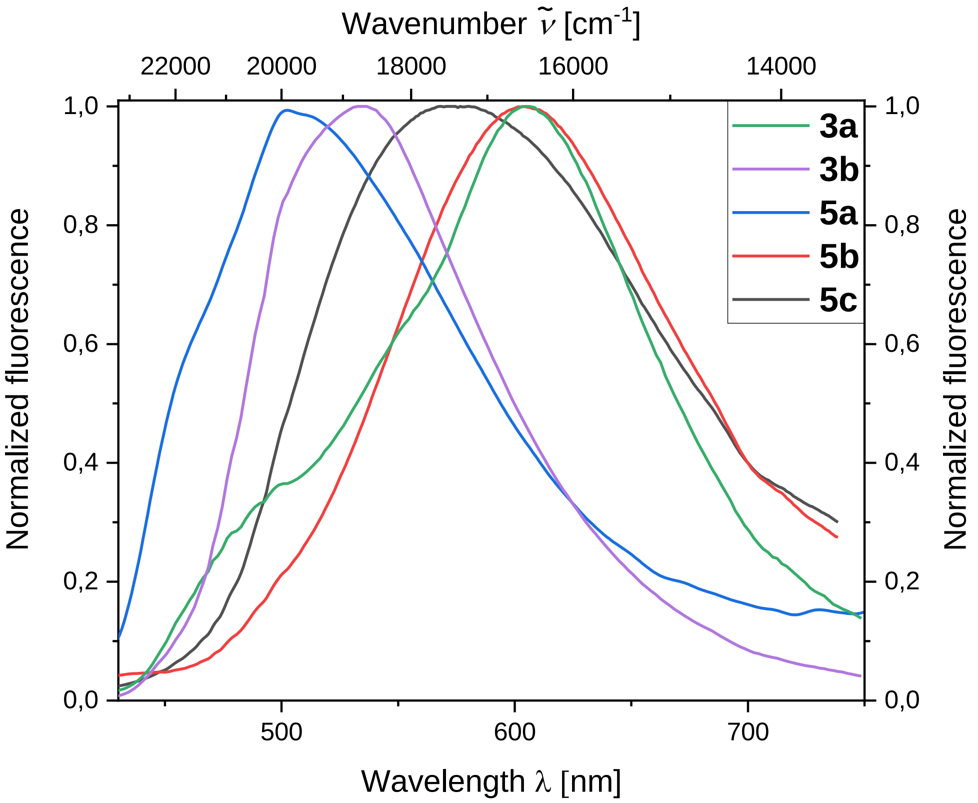

Figure S2: Normalized solid-state emission bands of aroyl-*S,N*-ketene acetal metal sensors **3** and **5**.

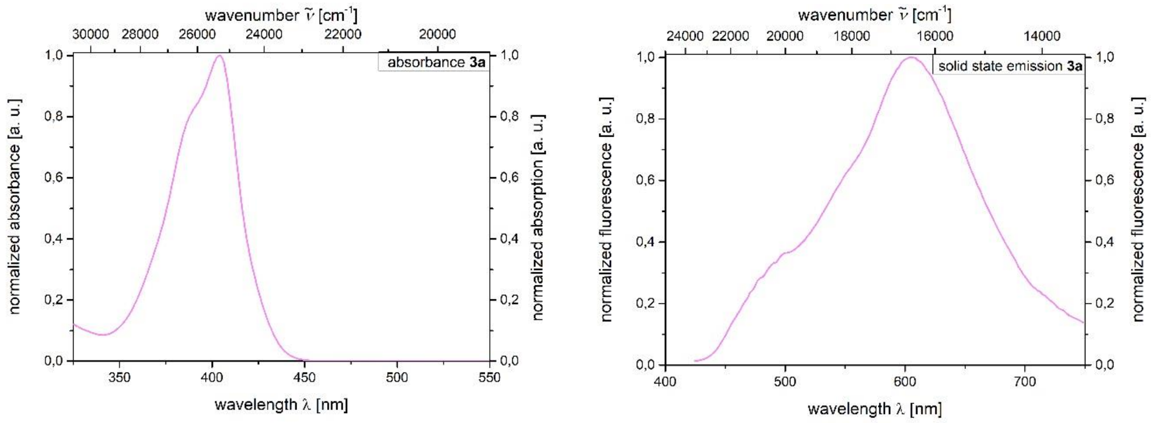

Figure S3: Absorption spectrum of compound **3a** in ethanol and solid-state emission spectrum of compound **3a**.

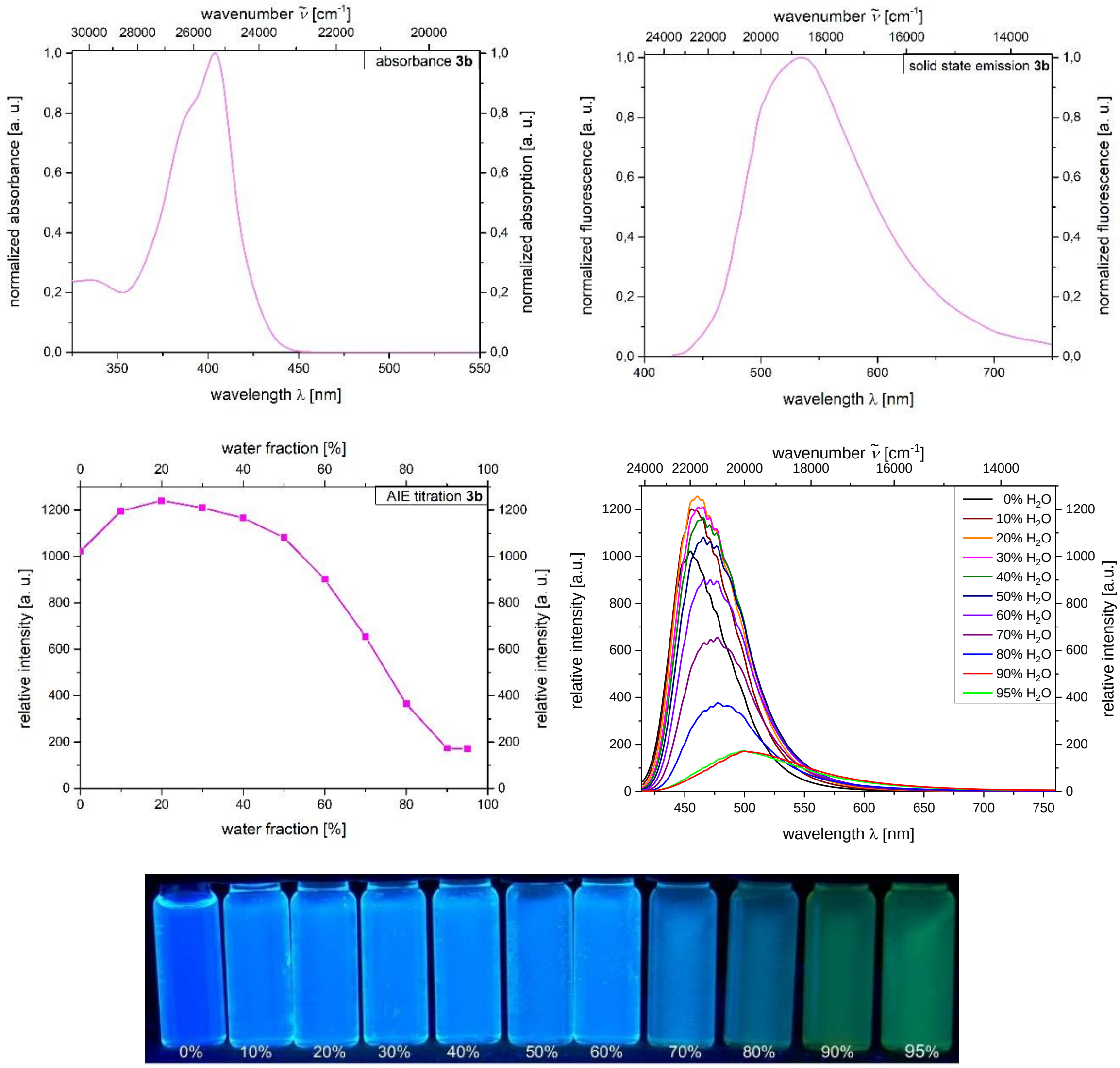

Figure S4: Absorption spectrum of compound **3b** in ethanol (top, left), solid state emission spectrum (top, right), and aggregation-induced changes in emission (center, left), aggregation-related emission spectra of compound **3b** (center, right), and photographs of solutions of dye **3b** in ethanol/water mixtures of increasing water content (bottom). The latter spectra were measured in ethanol/water mixtures of varying water content.

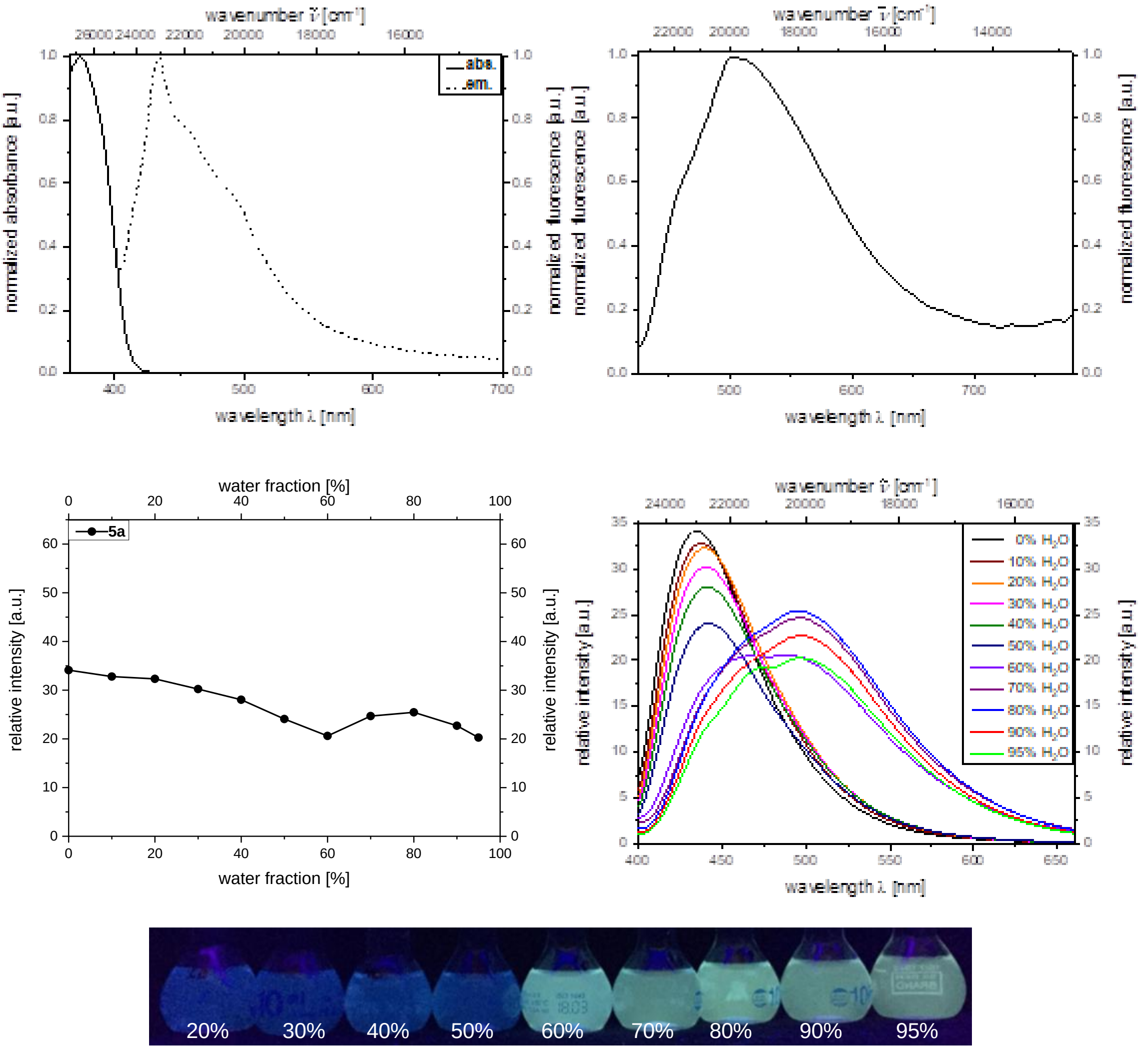

Figure S5: Absorption and emission spectrum of compound **5a** in ethanol (top, left), solid-state emission spectrum (top, right), and aggregation-induced changes in emission (center, left), aggregation-related emission spectra of compound **5a** (center, right) and photographs of solutions of dye **5a** in ethanol/water mixtures of increasing water content (bottom). The latter spectra were measured in ethanol/water mixtures of varying water content.

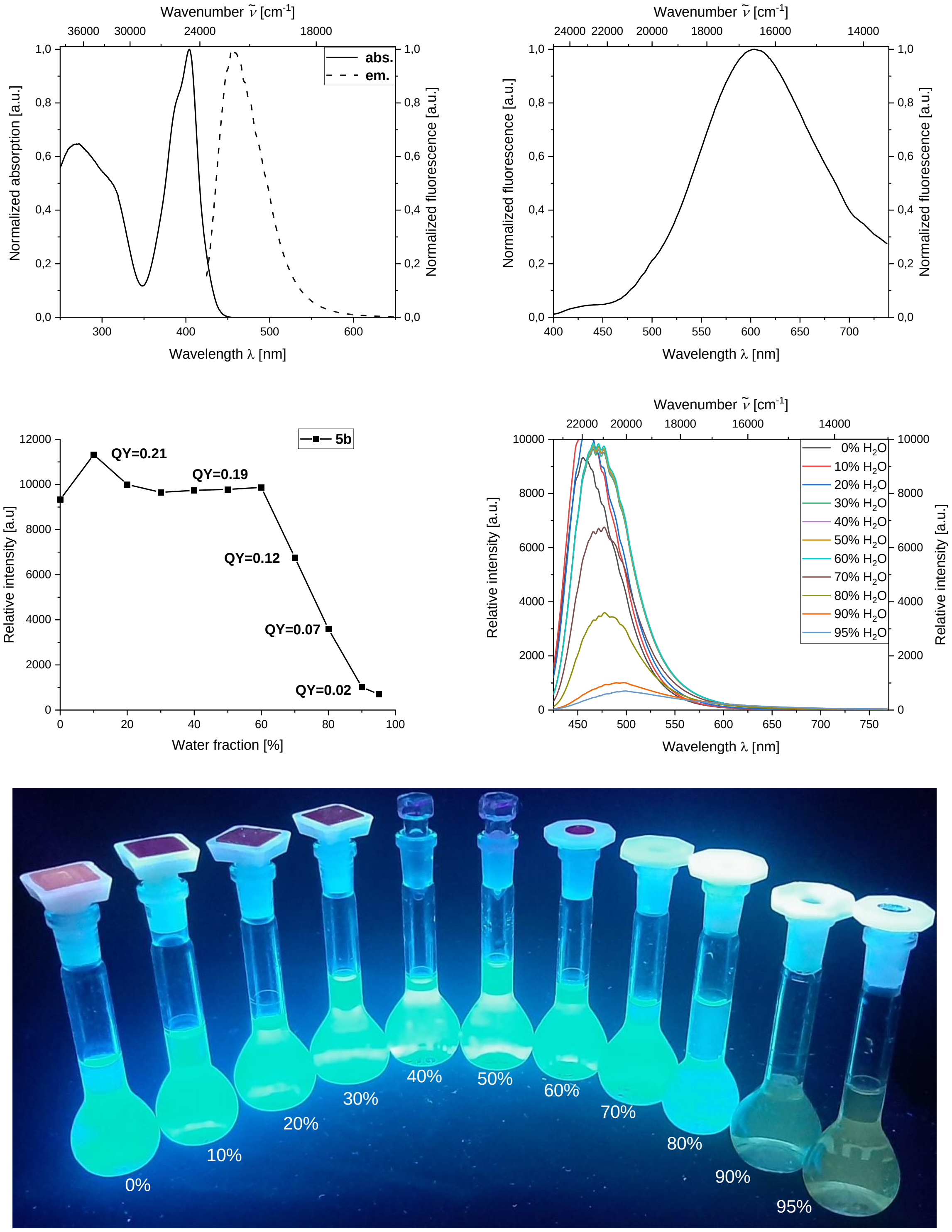

Figure S6: Absorption and emission spectrum of compound **5b** in ethanol (top, left), solid-state emission spectrum (top, right), and aggregation-induced changes in emission (center, left), aggregation-related emission spectra of compound **5b** (center, right) and photographs of solutions of dye **5b** in ethanol/water mixtures of increasing water content (bottom). The latter spectra were measured in ethanol/water mixtures of varying water content.

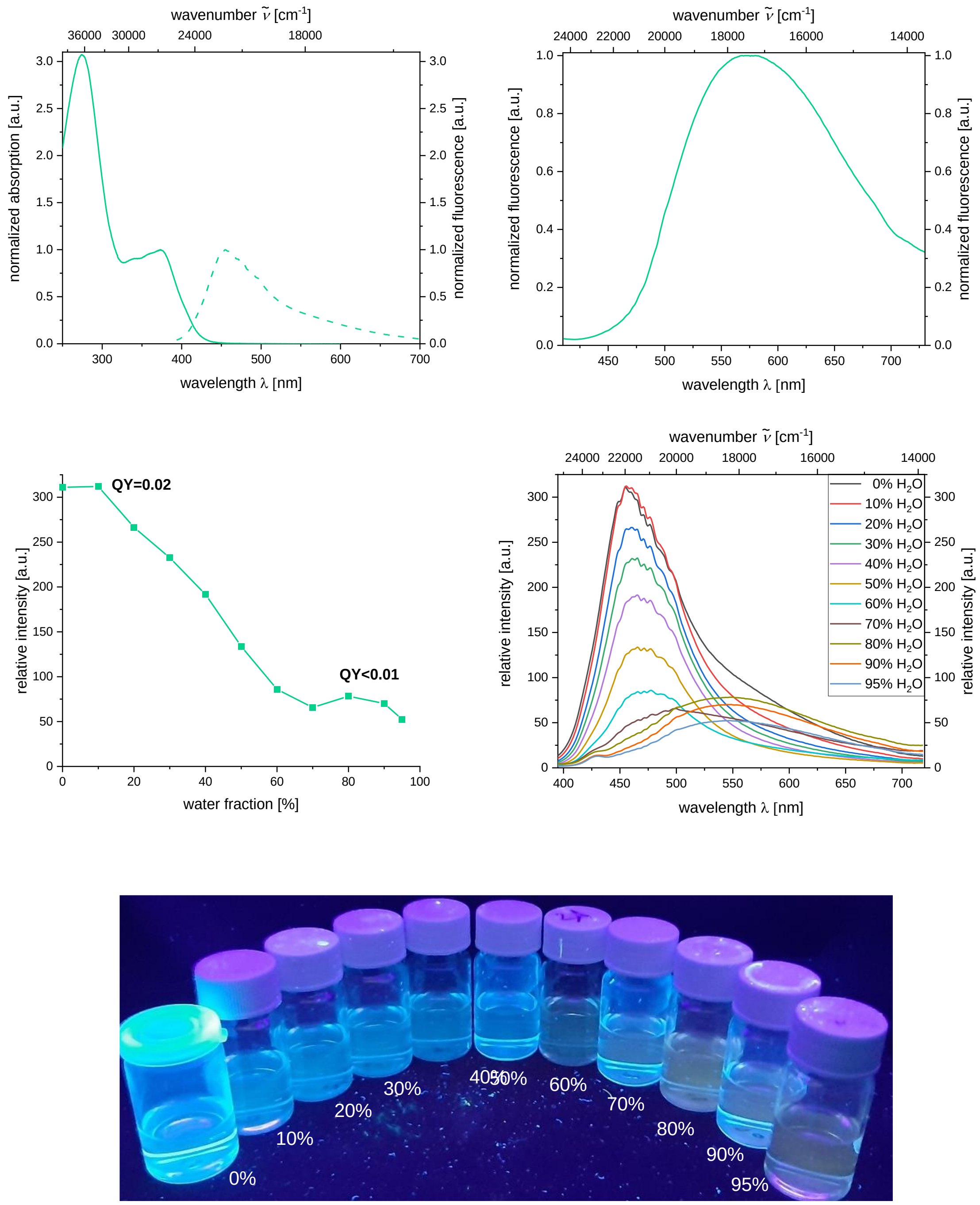

Figure S7: Absorption and emission spectrum of **5c** in ethanol (top, left), solid state emission spectrum (top, right), and aggregation-induced changes in emission (center, left), aggregation-related emission spectra of compound **5c** (center, right) and photographs of solutions of dye **5c** in ethanol/water mixtures of increasing water content (bottom). The latter spectra were measured in ethanol/water mixtures of varying water content.

8 Complexometry

By default, the determination of complex stoichiometry is carried out using the method of continuous variation, that of the job plot. Here, the assumption of a constant total volume and concentration of all components involved in the equilibrium applies. The continuous variation of the ratios of metal salt and ligand concentration allows the determination of the complex stoichiometry via the change of the physical quantity under consideration. For the required job plot, the variable photophysical quantity must be plotted against the mole fraction.

A series of qualitative tests were carried out by adding small amounts of various metal salts to a solution of the dye in ethanol and observing with the naked eye what influence this had on the absorption and emission properties of the dissolved chromophore.

Two criteria must be met for this method to deliver evaluable results: First, there must be a clearly defined complex stoichiometry and, second, a physically measurable quantity must be variable. In order to produce a Job plot, difference spectra must be prepared by subtracting the spectrum of the pure ligand from the spectrum of the ligand in the presence of the metal salt. The most prominent maxima or minima of the difference spectra can be used for the Job plot, as the corresponding signal-to-noise ratio is minimal. The position of the maximum or minimum, or possibly the maxima and minima, in the resulting Job plot determines the stoichiometry present in the complex. The abscissa at the maximum value of the ordinate determines the complex stoichiometry. If the maximum is below 0.5, the ligand content dominates, if the maximum is 0.5, a 1:1 stoichiometry can be assumed, if the abscissa value is above 0.5, a dominance of the metal in the complex can be assumed. The stability of the complex can be seen from the shape of the Job plot. Parabolic curves of the job plot indicate a less stable complex.; These can often be approximated by quadratic or even-numbered equations. If, on the other hand, there are two approximately linear subsegments with an intersection point, a stable complex can be assumed. It should also be noted that free binding sites in the complex can be occupied by solvent molecules if coordinating solvents are present.

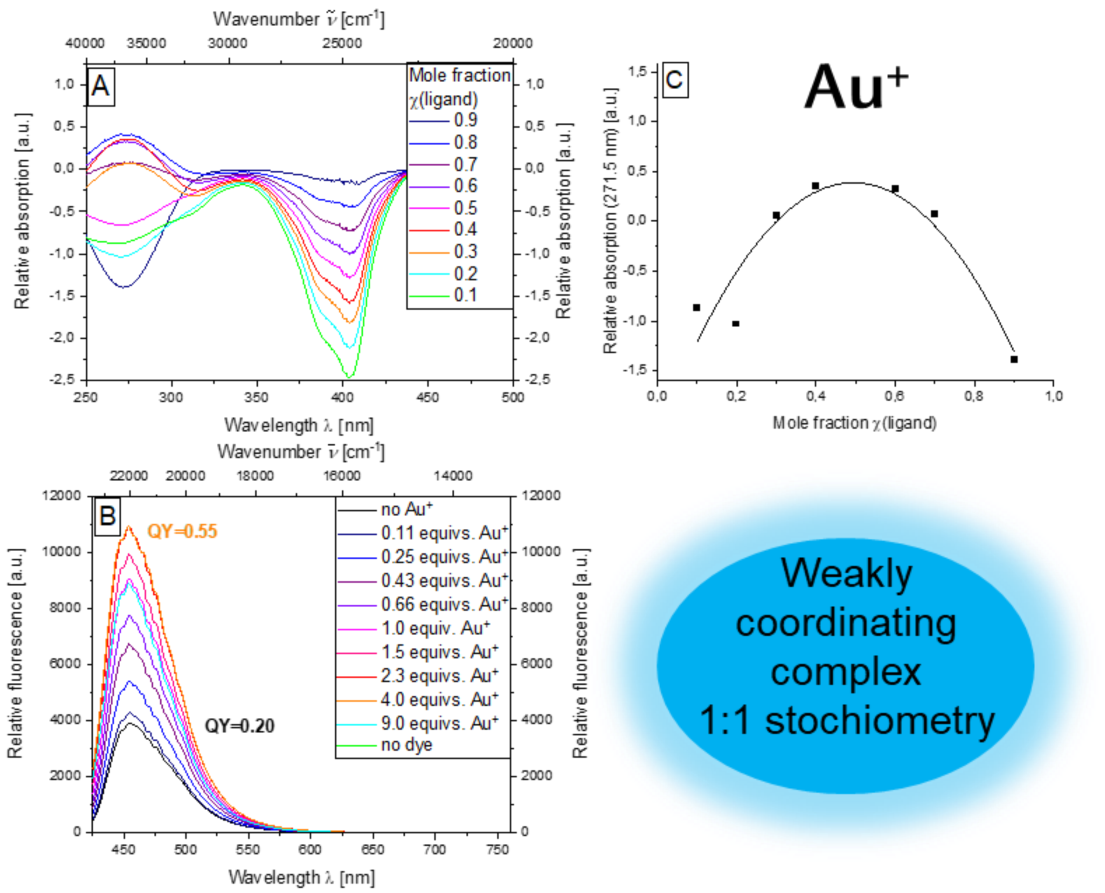

Figure S8: Absorption difference spectra of compound **3b** at different mole fractions of the ligating dye in the presence of AuI (recorded in ethanol; *c*(**3b**) = 10^{-5} M, *c*(AuI) = 10^{-5} M, *T* = 298 K) (**A**), emission spectra of compound **3b** with increasing amount AuI (*c*(**3b**) = 10^{-7} M, *c*(AuI) = 10^{-7} M, *T* = 298 K, λ_{exc} = 404 nm) (**B**), *Job* plot of the mole fraction of the ligand against the relative absorption at 271.5 nm (**C**).

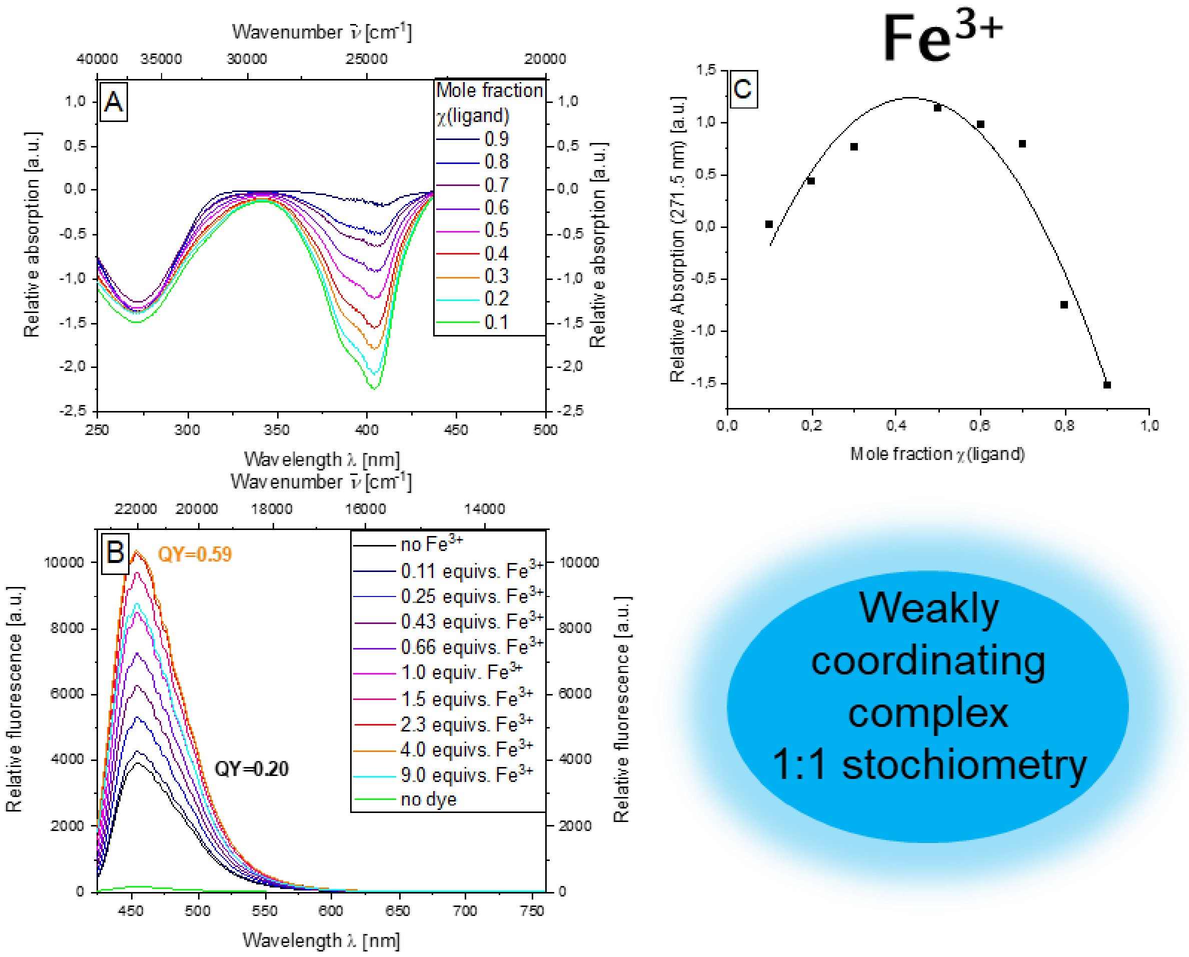

Figure S9: Absorption difference spectra of compound **3b** at different mole fractions of the ligating dye in the presence of $FeCl_3$ (recorded in ethanol; c(**3b**) = 10^{-5} M, $c(FeCl_3)$ = 10^{-5} M, T = 298 K) (**A**), emission spectra of compound **3b** with increasing amount $FeCl_3$ (c(**3b**) = 10^{-7} M, $c(FeCl_3)$ = 10^{-7} M, T = 298 K, λ_{exc} = 404 nm) (**B**), *Job* plot of the mole fraction of the ligand against the relative absorption at 271.5 nm (**C**).

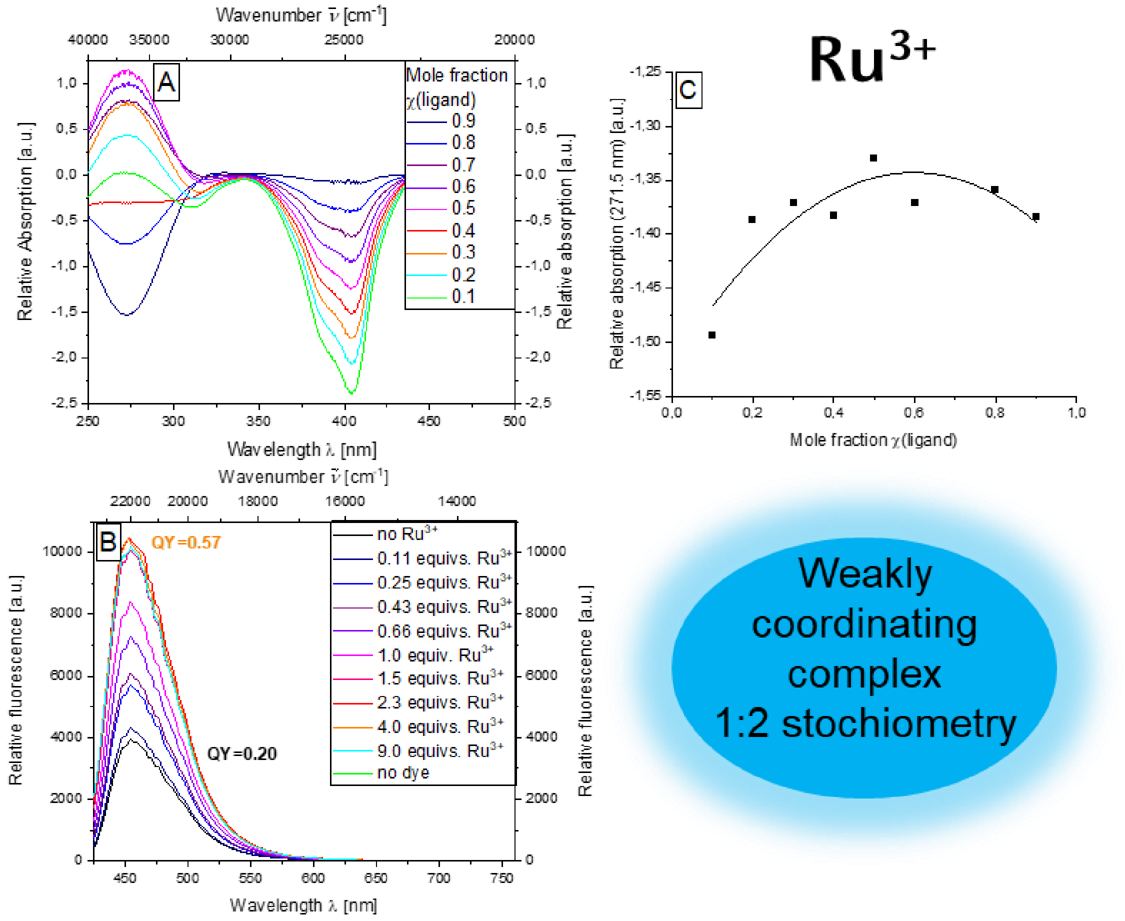

Figure S10: Absorption difference spectra of compound **3b** at different mole fractions of the ligating dye in the presence of $RuCl_3$ (recorded in ethanol; c(**3b**) = 10^{-5} M, $c(RuCl_3)$ = 10^{-5} M, T = 298 K) (**A**), emission spectra of compound **3b** with increasing amount $RuCl_3$ (c(**3b**) = 10^{-7} M, $c(RuCl_3)$ = 10^{-7} M, T = 298 K, λ_{exc} = 404 nm) (**B**), *Job* plot of the mole fraction of the ligand against the relative absorption at 271.5 nm (**C**).

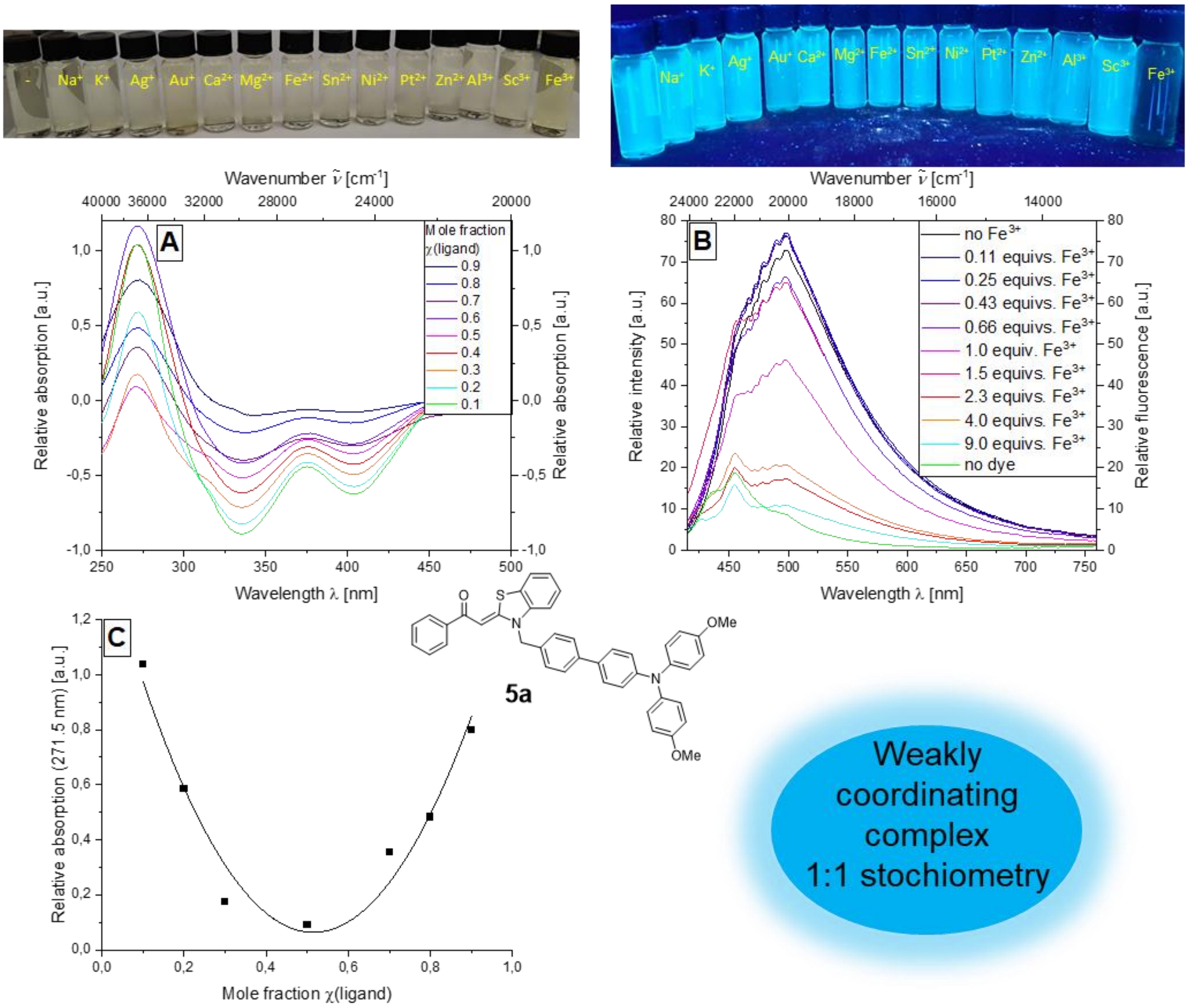

Figure S11: Top: apparent perception of the properties of **5a** when different metal salts are added in daylight (left) and under UV light (right); center: absorption difference spectra of compound **5a** at different mole fractions of the ligating dye in the presence of $FeCl_3$ (recorded in ethanol; *c*(**5a**) = 10^{-5} M, *c*($FeCl_3$) = 10^{-5} M, *T* = 298 K) (**A**), Emission spectra of compound **5a** with increasing amount $FeCl_3$ (*c*(**5a**) = 10^{-7} M, *c*($FeCl_3$) = 10^{-7} M, *T* = 298 K, λ_{exc} = 373 nm) (**B**); bottom: *Job* plot of the mole fraction of the ligand against the relative absorption at 271.5 nm (**C**).

Ru^{3+}

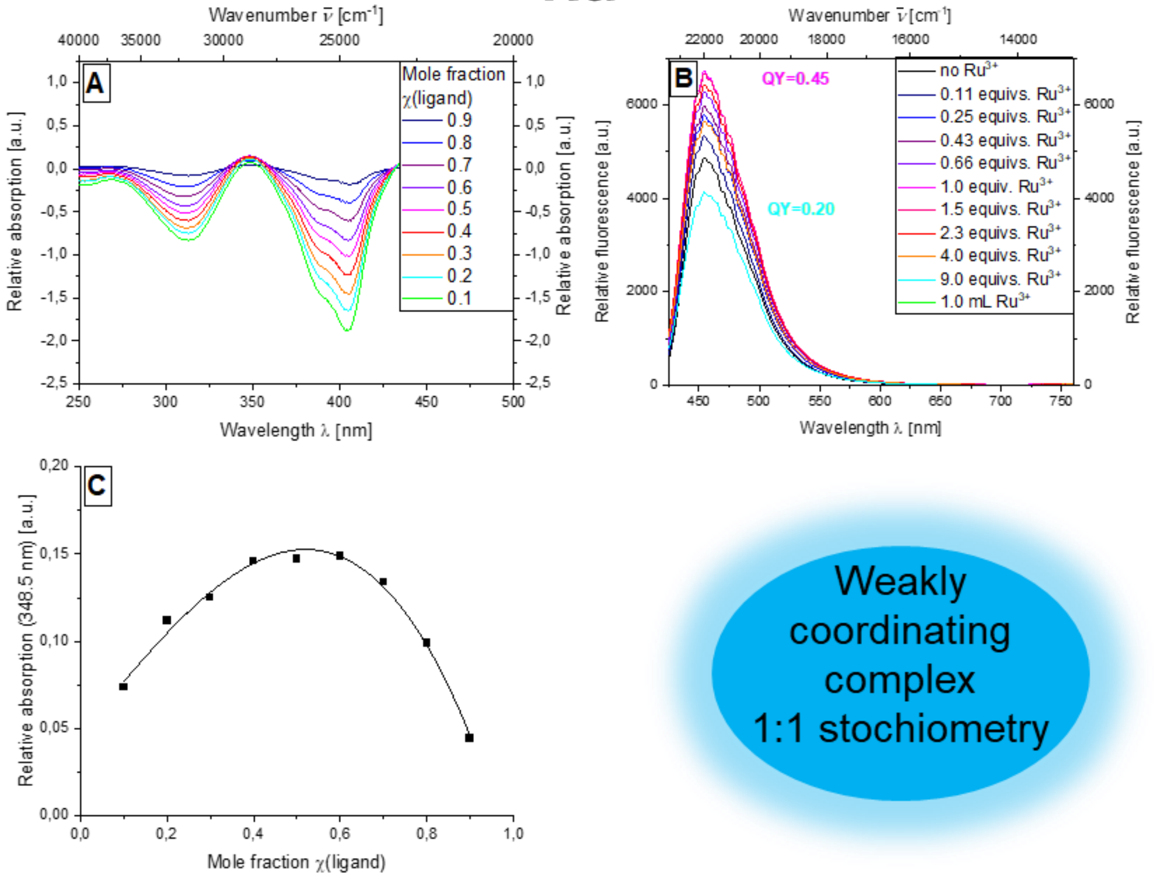

Figure S12: Absorption difference spectra of compound **5b** at different mole fractions of the ligating dye in the presence of $RuCl_3$ (recorded in ethanol; c(**5b**) = 10^{-5} M, $c(RuCl_3) = 10^{-5}$ M, T = 298 K) (**A**), emission spectra of compound **5b** with increasing amount $RuCl_3$ (c(**5b**) = 10^{-7} M, $c(RuCl_3) = 10^{-7}$ M, T = 298 K, λ_{exc} = 404 nm) (**B**), *Job* plot of the mole fraction of the ligand against the relative absorption at 348.5 nm (**C**).

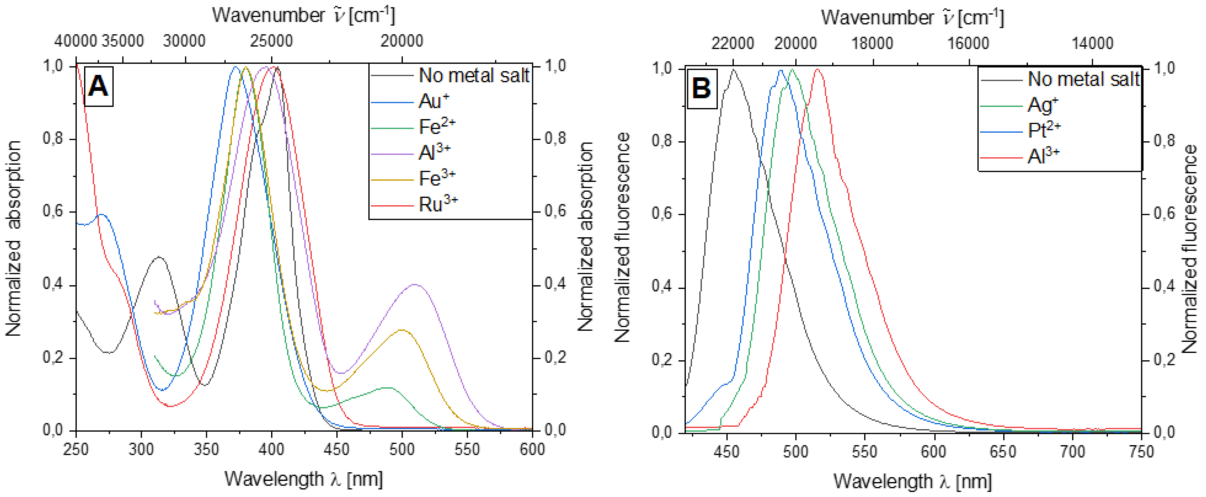

Figure S13: Absorption spectra on addition of various metal salts in ethanol (c(**5b**) = 10^{-5} M, $c(M^+)$ = 10^{-5} M, T = 298 K) (**A**) and emission spectra of compound **5b** on addition of various metal salts in ethanol (c(**5b**) = 10^{-7} M, $c(M^+)$ = 10^{-7} M, T = 298 K, λ_{exc} = 404 nm) (**B**).

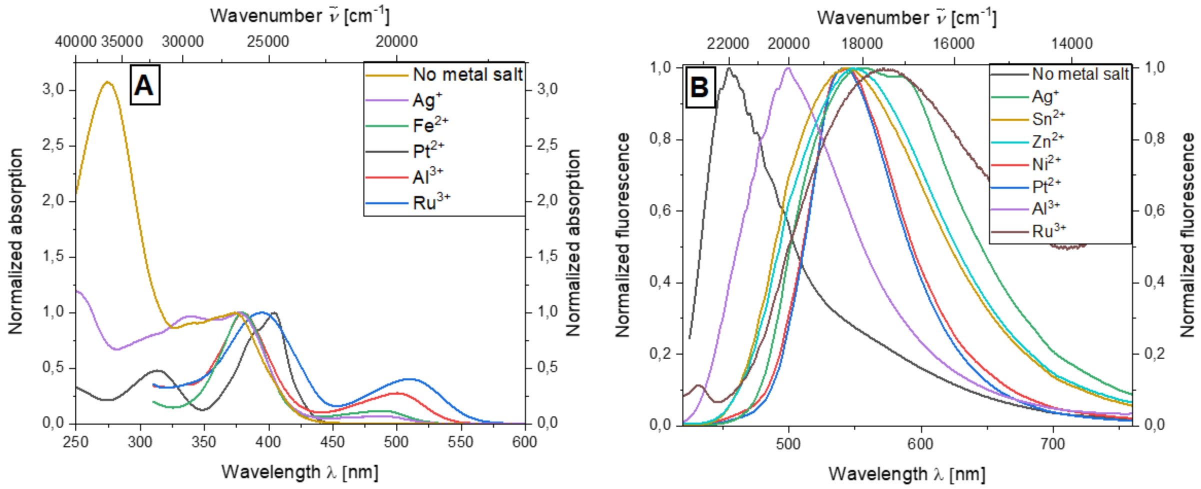

Figure S14: Absorption spectra on addition of various metal salts in ethanol (c(**5c**) = 10^{-5} M, $c(M^+)$ = 10^{-5} M, T = 298 K) (**A**) and emission spectra of compound **5c** on addition of various metal salts in ethanol (c(**5c**) = 10^{-7} M, $c(M^+)$ = 10^{-7} M, T = 298 K, λ_{exc} = 404 nm) (**B**).

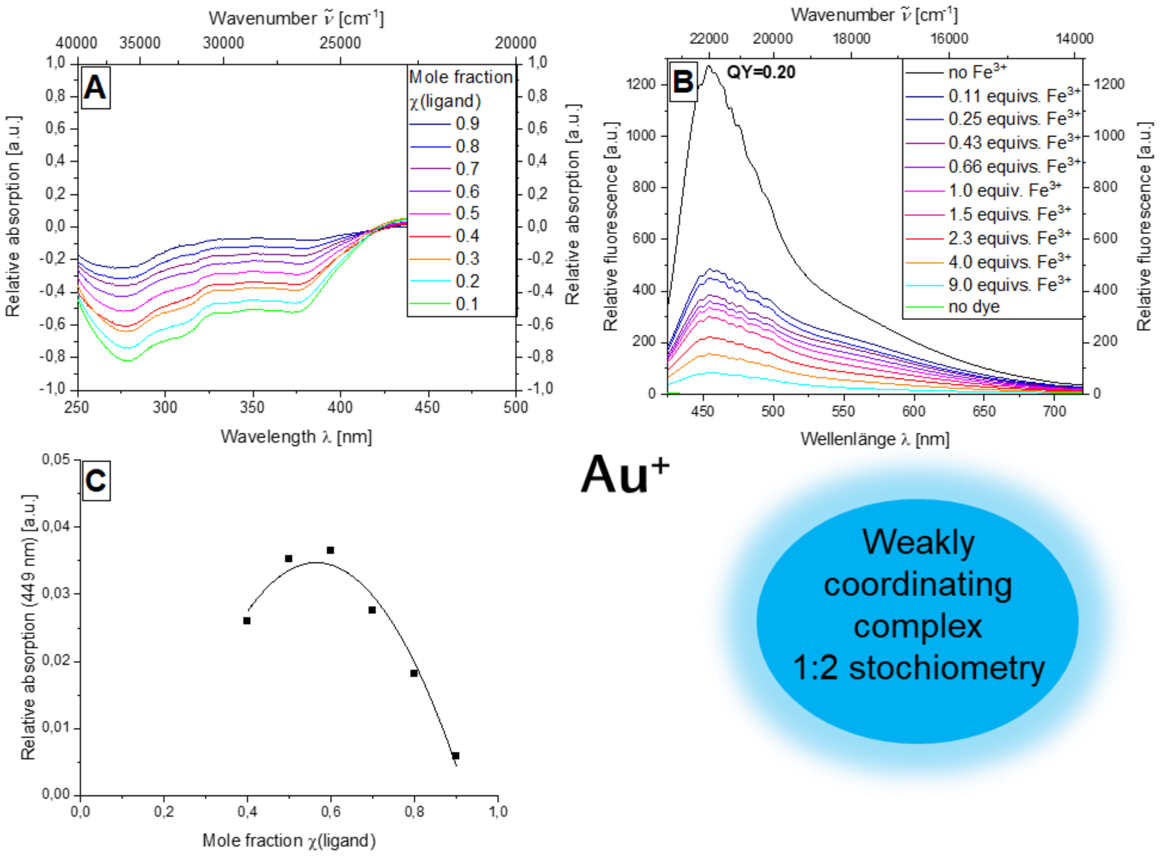

Figure S15: Absorption difference spectra of compound **5c** at different mole fractions of the ligating dye in the presence of AuI (recorded in ethanol; c(**5c**) = 10^{-5} M, c(AuI) = 10^{-5} M, T = 298 K) (**A**), emission spectra of compound **5c** with increasing amount AuI (c(**5c**) = 10^{-7} M, c(AuI) = 10^{-7} M, T = 298 K, λ_{exc} = 404 nm) (**B**), *Job* plot of the mole fraction of the ligand against the relative absorption at 449 nm (**C**).

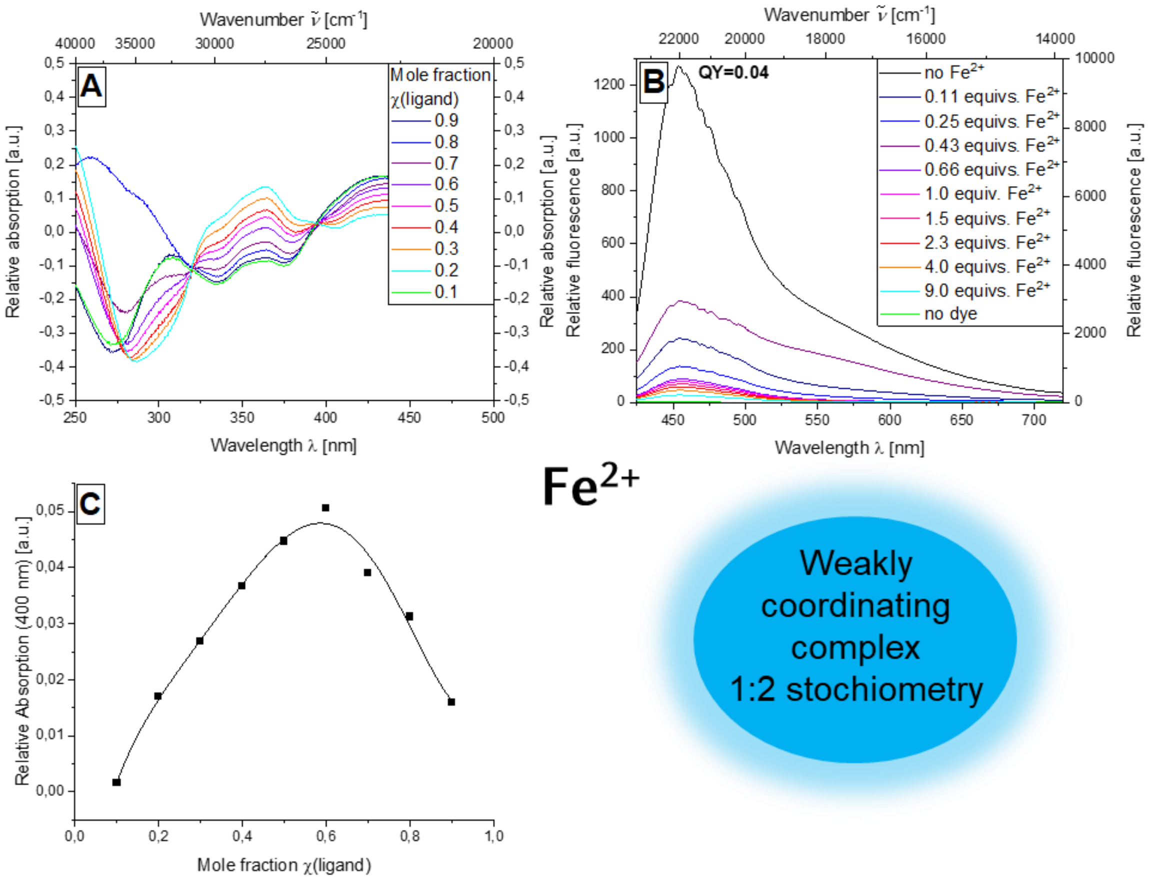

Figure S16: Absorption difference spectra of compound **5c** at different mole fractions of the ligating dye in the presence of $FeSO_4$ (recorded in ethanol; *c*(**5c**) = 10^{-5} M, *c*($FeSO_4$) = 10^{-5} M, T = 298 K) (**A**), emission spectra of compound **5c** with increasing amount $FeSO_4$ (*c*(**5c**) = 10^{-7} M, *c*($FeSO_4$) = 10^{-7} M, T = 298 K, λ_{exc} = 404 nm) (**B**), *Job* plot of the mole fraction of the ligand against the relative absorption at 400 nm (**C**).

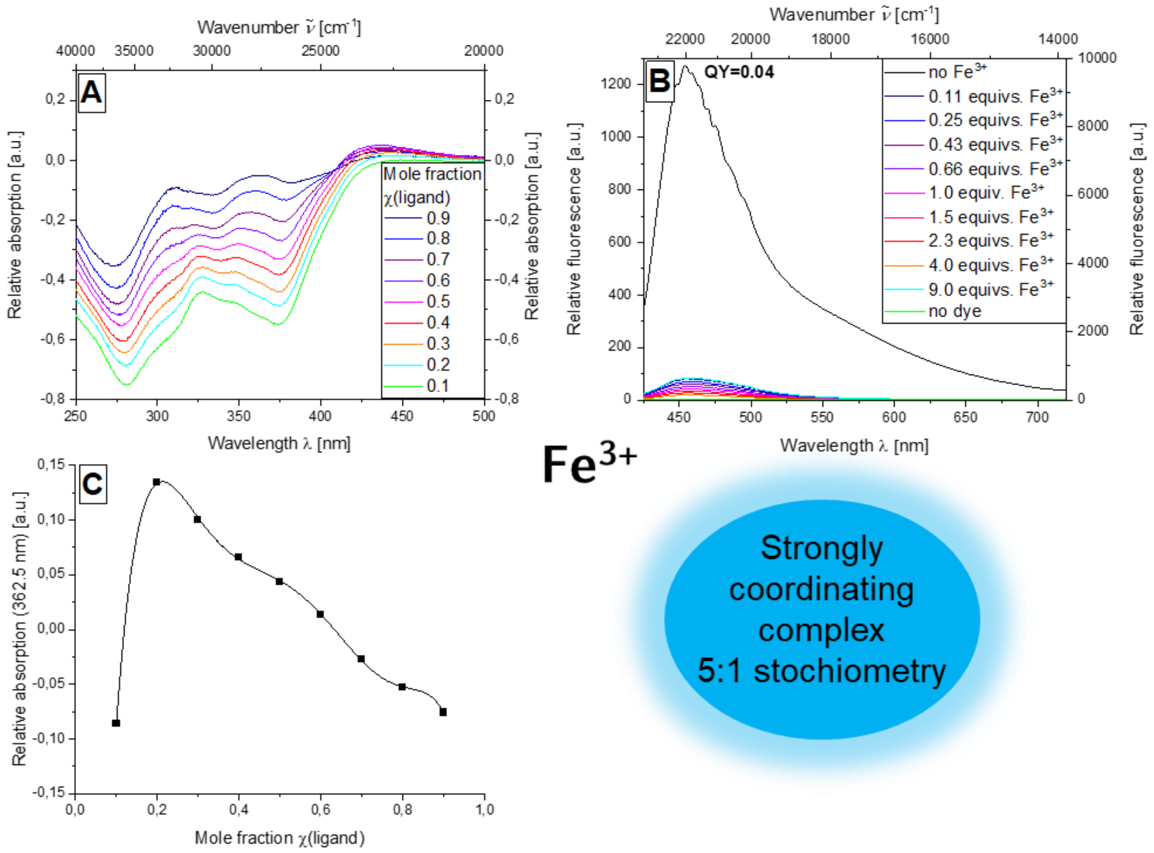

Figure S17: Absorption difference spectra of compound **5c** at different mole fractions of the ligating dye in the presence of $FeCl_3$ (recorded in ethanol; *c*(**5c**) = 10^{-5} M, *c*($FeCl_3$) = 10^{-5} M, *T* = 298 K) (**A**), emission spectra of compound **5c** with increasing amount $FeCl_3$ (*c*(**5c**) = 10^{-7} M, *c*($FeCl_3$) = 10^{-7} M, *T* = 298 K, λ_{exc} = 404 nm) (**B**), *Job* plot of the mole fraction of the ligand against the relative absorption at 362.5 nm (**C**).
